# Yunvjian decoction attenuates lipopolysaccharide-induced acute lung injury by inhibiting NF-κB/NLRP3 pathway and pyroptosis

**DOI:** 10.3389/fphar.2025.1430536

**Published:** 2025-01-24

**Authors:** Fanxuan Zhang, Fang Wang, Lisha Zhao, Leqian Wang, Wenjing Li, Feihua Huang, Nani Wang

**Affiliations:** ^1^ Second Clinical Medical College, Zhejiang Chinese Medical University, Hangzhou, Zhejiang, China; ^2^ School of Pharmacy, Zhejiang Chinese Medical University, Hangzhou, Zhejiang, China; ^3^ Tongde Hospital, Zhejiang Chinese Medical University, Hangzhou, Zhejiang, China; ^4^ College of Pharmacy, Hangzhou Normal University, Hangzhou, Zhejiang, China

**Keywords:** Yunvjian decoction, acute lung injury, active ingredients, NF-κB, NLRP3

## Abstract

**Introduction:**

Yunvjian (YNJ) decoction, a classic traditional Chinese medicine prescription for inflammatory diseases, has demonstrated good therapeutic effects in the clinical treatment of pneumonia. The aim of this study was to clarify the effective ingredients and mechanism of action of YNJ on lipopolysaccharide (LPS)-induced acute lung injury (ALI).

**Methods:**

The effects of YNJ were evaluated in a mouse model of LPS-induced ALI and in LPS-treated MLE-12 murine lung epithelial cells and RAW264.7 macrophages *in vitro*. The mechanism of action of YNJ on these model systems was studied using RNA sequencing, immunohistochemical analysis, immunoblotting, immunofluorescence, ELISA, and polymerase chain reaction assays. Ultra-high performance liquid chromatography coupled with quadrupole time-of-flight mass spectrometry was applied to identify the absorbed components of YNJ.

**Results:**

YNJ attenuated pulmonary damage in LPS-treated mice, as evidenced by reduced protein content in bronchoalveolar lavage fluid, decreased lung wet/dry weight ratio, and improved respiratory function. Analysis of pneumonia-related lung injury samples from patients in the Gene Expression Omnibus dataset GSE40012 indicated that NOD-like receptor protein 3 (NLRP3)-mediated pyroptosis was a primary mechanism in ALI. YNJ reduced the phosphorylation of nuclear factor-kappa B (NF-κB) and decreased the expression levels of lung NLRP3, apoptosis-associated speck-like protein containing a CARD (ASC), cleaved caspase-1, and interleukin-1β levels (IL-1β) *in vivo*. Administration of YNJ-containing mouse serum increased cell viability and decreased malondialdehyde and reactive oxidative species contents in LPS-stimulated MLE-12 cells. YNJ-containing serum also decreased the secretion of tumor necrosis factor-α, IL-6, and IL-1β in LPS-stimulated RAW264.7 macrophages, and promoted macrophage polarization toward an M2 phenotype. A total of 23 absorbed components were identified in YNJ-containing serum. Among those, network analysis and *in vitro* experiments indicated that diosgenin, timosaponin BII, and mangiferin are anti-inflammatory active substances.

**Conclusion:**

YNJ attenuates LPS-induced ALI in mice by inhibiting pyroptosis of lung epithelial cells and macrophages via suppression of the NF-κB/NLRP3 pathway. Our findings provide novel insights into the therapeutic effects of YNJ on ALI.

## 1 Introduction

Acute lung injury (ALI) is a common respiratory disease caused by sepsis, pneumonia associated with bacterial, fungal, or viral infection, mechanical impact, acute pancreatitis, and other noxious stimuli. ALI primarily manifests as edema, an excessive inflammatory response triggered by immune cell infiltration (Feng et al., 2017), and oxidative stress damage (Aziz et al., 2018). The alveolar epithelium plays a crucial role in maintaining lung structure and function by defending against microorganisms and small particles (Georas and Rezaee, 2014). Diffuse alveolar epithelial cell injury and disruptions in epithelial barrier integrity are critical in the occurrence and development of ALI (Lee et al., 2018; Thompson et al., 2017). Macrophages are the primary barrier against foreign invasion, participating in extracellular matrix remodeling, angiogenesis, and inflammation (Cheng et al., 2021). Macrophages also directly promote epithelial cell proliferation and drive epithelial tissue repair (Puttur et al., 2019; Hung et al., 2019). Currently, symptomatic therapy, such as mechanical ventilation and fluid management, is commonly used for treating ALI (Liu et al., 2022). Given the poor effectiveness of hormonal therapy during early ALI, treatment options for early ALI remain limited.

Pyroptosis is a form of regulated cell death that is accompanied by the release of active proinflammatory cytokines (Rao et al., 2022) and plays an important role in the development of ALI (Liu B. et al., 2021). The initiation of pyroptosis is triggered by activation of the NOD-like receptor family pyrin domain containing 3 (NLRP3) inflammasome (Feng Y. et al., 2022; Freeman and Swartz, 2020). NLRP3 stimulation contributes to ALI pathogenesis by inducing the recruitment of apoptosis-associated speck-like protein containing a CARD (ASC) and pro-caspase-1, leading to the maturation of the cytokine interleukin-1β (IL-1β) to activate the inflammatory cascade (Peng et al., 2020; Danielski et al., 2020). Nuclear factor-kappa B (NF-κB), a transcription factor that regulates the expression of various proinflammatory cytokines, acts as an upstream activator of NLRP3 (Jiang et al., 2016). Thus, the NF-κB/NLRP3 pathway is considered an essential target for ALI treatment.

Yunvjian decoction (YNJ) is a classic traditional Chinese medicine prescription. It contains five constituents: gypsum, *Rehmannia glutinosa* (Gaertn.) D.C., *O. japonicus* (Thunb.) Ker Gawl., *Anemarrhena asphodeloides* Bunge, and *A. bidentata* Blume (Ye et al., 2024). Past research has reported the beneficial effects of YNJ components on lung disease. The extract of *Rehmannia glutinosa* (Gaertn.) DC. mitigated lipopolysaccharide (LPS)-induced inflammatory cell infiltration and the production of IL-1β and interleukin-6 (IL-6), thereby reducing lung inflammation and improving lung function (Jing et al., 2015). *Ophiopogon japonicus* (Thunb.) Ker Gawl. attenuated the inflammatory response and fibrosis in the lungs of radiation-treated mice (Yao et al., 2019). Ethanol extract of *Anemarrhena asphodeloides* Bunge reduced inflammatory cell infiltration in the bronchoalveolar lavage fluid (BALF) of LPS-induced ALI mice (Park et al., 2018). However, there are few reports on the mechanisms of action of YNJ on ALI.

To address this research gap, in this study a mouse model of LPS-induced ALI, murine lung epithelial cell-12 (MLE-12) cells, and macrophage models were used to study the therapeutic mechanisms of YNJ on ALI, with particular focus on the potential regulation of the NF-κB/NLRP3 pathway and pyroptosis.

## 2 Materials and methods

### 2.1 YNJ preparation

YNJ was obtained from Tongde Hospital of Zhejiang Province (Batch No: 230530, Hangzhou, China). YNJ is composed of 14.92 g of gypsum (specimen number #220103), 24.25 g of *Rehmannia glutinosa* (Gaertn.) DC. (specimen number #220116), 7.46 g of *Ophiopogon japonicus* (Thunb.) Ker Gawl. (specimen number #211008), 5.60 g of *Anemarrhena asphodeloides* Bunge. (specimen number #211231), and 5.60 g of *Achyranthes bidentata* Blume (specimen number #220207). All drugs were purchased from Zhejiang Tongjuntang Herbal Pieces Co., Ltd. (Zhejiang, China). YNJ was extracted with water (1:8, w/v) at 100°C for 2 h. The extracts were lyophilized and stored at 4°C until use.

### 2.2 Animals

Male C57BL/6 mice (8 weeks old) were obtained from Hangzhou Medical College (Zhejiang, China). Mice were maintained at a constant temperature (24°C–26°C) and humidity (30%–50%) with a 12 h light/dark cycle. Animals were allowed free access to food and water. Mice were randomized into six groups: 1) Control group (normal saline, intranasal inhalation (i.n.); normal saline, intragastric irrigation (i.g.)); 2) LPS group (LPS, #L8880, Solarbio, Beijing, China; 0.25 mg/kg/d, i.n.; normal saline, i.g.), 3–5) LPS + YNJ low, medium, and high (L, M, H) groups (LPS, 0.25 mg/kg/d, i.n.; 10 (YNJ-L), 20 (YNJ-M), and 40 (YNJ-H) g/kg/d YNJ, i.g.); and 6) LPS + amoxicillin (AMO) group (LPS, 0.25 mg/kg/d, i.n.; AMO; #470230109, Shijiazhuang Chino Pharmaceutical Group Pharmaceutical, Hebei, China; 0.78 g/kg/d, i.g.). The ALI model was constructed according to previous literature (Du et al., 2020; Wang Y. et al., 2022; Ehrentraut et al., 2019). The doses of YNJ and AMO were selected according to clinical practice and previous literature (Luo et al., 2023; Soliman et al., 2022). After treatment for 14 days, the mice were anesthetized and lung tissue, serum, and BALF were collected.

### 2.3 Pulmonary function measurement

Pulmonary function was examined according to previously described methods (Hashimoto et al., 2016). At the end of the treatment, the mice were mechanically ventilated with an electrophysiology instrument (#MP150, BIOPAC, United States). The peak inspiratory flow, peak expiratory flow, tidal volume, minute volume, total breathing time, and breathing rate were recorded.

### 2.4 Lung wet/dry weight (W/D) ratio

For lung W/D ratio estimations, the surface layer of the upper lobe of the right lung was wiped dry and then weighed. Subsequently, the lung tissue was dried in an oven at 80°C for 3 days and weighed (Shang et al., 2024).

### 2.5 Determination of protein concentration in BALF

BALF was obtained according to previous reports (Li et al., 2016). The collected BALF was centrifuged at 700 × g for 10 min, and the supernatant used for measurement of total protein levels using a BCA protein assay kit (#KGB2101, KeyGen, Jiangsu, China).

### 2.6 Hematoxylin-eosin (HE) staining

The middle and lower lobes of the right lung were harvested and fixed in 10% buffered formaldehyde solution for 24 h before being paraffin-embedded. HE staining was carried out on lung sections (5 μm thick) according to a standard protocol (Liu Z. et al., 2021) using an HE assays kit (#G1076, Servicebio, Hubei, China).

### 2.7 RNA sequencing (RNA-seq) analysis

RNA was isolated from lung tissue using TRIzol reagent (#G3013, Servicebio). Library quality was assessed on a high-throughput nucleic acid protein analysis system (#Qsep400, Houze Biotechnology, Zhejiang, China). cDNA fragments with a length of 150 ∼ 200 bp were selected for Polymerase chain reaction (PCR) and purified, and the quality of the library was evaluated. Cluster analysis of RNA-seq data was carried out and results visualized by heatmaps and volcano plots.

### 2.8 Clinical bioinformatics

We mined the Gene Expression Omnibus database (GSE40012, http://www.ncbi.nlm.nih.gov/geo/), to identify differentially expressed genes (DEGs) between healthy and pulmonary inflammatory patient samples. A *P* value <0.01 and a threshold value ≥1.5 for fold change |FC| were applied.

### 2.9 Immunohistochemical (IHC) staining

IHC staining (Shang et al., 2024) was conducted to detect the expression of p-NF-κB (1:200; #AF2006, Affinity Biosciences, Jiangsu, China), NLRP3 (1:200; #DF7438, Affinity Biosciences), ASC (1:200; #DF6304, Affinity Biosciences) and cleaved caspase-1 (1:200; #AF4022, Affinity Biosciences) in lung tissue.

### 2.10 Preparation of YNJ-containing serum

Male C57BL/6 mice were randomly divided into two groups: the YNJ serum group and the blank serum group. The mice in the YNJ serum group were orally administered YNJ (40 g/kg/d). The mice in the blank serum group were treated with an equal volume of distilled water. The mice were treated for 7 days (Ren et al., 2023). One hour after the last treatment, blood samples were collected and centrifuged at 3,000 × g for 15 min. The supernatant was inactivated at 56°C for 30 min. The bacteria were removed by a 0.22-μm filtration membrane. The YNJ-containing serum was stored at −80°C for subsequent experiments.

### 2.11 Cell culture

MLE-12 mouse lung epithelial cells (#JY106, Jingyuan Biotechnology, Shanghai, China) and RAW264.7 macrophages (#CBP60533, National Collection of Authenticated Cell Cultures, Shanghai, China) were maintained in Dulbecco’s modified Eagle’s medium supplemented with 10% fetal bovine serum. MLE-12 and RAW264.7 cells were respectively divided into six groups: 1) a blank control group; 2) LPS group (500 ng/mL LPS); 3) YNJ group (YNJ-containing serum +500 ng/mL LPS); 4) nigericin (#HY-127019, MedchemExpress) + YNJ group (10 μM nigericin +2% YNJ-containing serum +500 ng/mL LPS); 5) LPS/adenosine 5′-triphosphate (ATP; #HY-B2176, MedchemExpress) group (500 ng/mL LPS +5 mM ATP); and 6) LPS/ATP + YNJ group (500 ng/mL LPS +5 mM ATP +2% YNJ-containing serum). After 24 h-treatment, the cell proliferation rate was measured (Cao et al., 2022; Tang et al., 2019). For verify the activity of the compounds, cells were divided into blank control group, LPS group (500 ng/mL LPS), diosgenin (#180911, Aoke biosciences) group (500 ng/mL LPS +0.1 μM diosgenin), timosaponin BII (#A10307, Yuanye biosciences) group (500 ng/mL LPS +0.1 μM timosaponin BII), mangiferin (#B01367, Yongjian pharmaceutical) group (500 ng/mL LPS +0.1 μM mangiferin) and caffeic acid (#A10056, Yuanye biosciences) group (500 ng/mL LPS +0.1 μM caffeic acid).

### 2.12 Cell viability assay

MLE-12 cells RAW264.7 macrophages were incubated with 3-(4,5-dimethylthiazol-2-yl)-2,5-diphenyltetrazolium bromide (MTT, #KGA311, KeyGen) at 37°C for 4 h. Dimethyl sulfoxide was added to dissolve the produced formazan salts. Absorbance was measured at 490 nm using a microplate reader (#Spectra MAX 190, Molecular Devices, California, United States).

### 2.13 Measurement of malondialdehyde (MDA) levels

MDA levels were measured in MLE-12 cells using an MDA assay kit (#S0131, Beyotime, Shanghai, China). Absorbance was recorded at 450 nm by a microplate reader.

### 2.14 Detection of intracellular reactive oxygen species (ROS) levels

For ROS detection, MLE-12 cells were incubated for 20 min in serum-free culture medium containing 10 μM 2,7-dichlorofluorescein diacetate (#S0033, Beyotime) in the dark. Following cell harvesting with trypsin, fluorescence intensities were measured using flow cytometry (DxFLEX system, Beckman Coulter, California, United States).

### 2.15 Measurement of tumor necrosis factor-α (TNF-α), IL-6, and IL-1β levels

The secretion of TNF-α, IL-6, and IL-1β by cultured RAW264.7 macrophages was determined by ELISA using commercial kits (#KE1002, #KE1007, and #KE1003, Proteintech, Hubei, China). The absorbance was recorded at 450 nm by a microplate reader.

### 2.16 PCR assay

Total RNA was extracted using TRIzol reagent. Subsequently, cDNA synthesis was carried out using a reverse transcription kit (#G3329, Servicebio) and PCR conducted under standard conditions. Agarose gel electrophoresis was used for analysis of PCR products (Quang et al., 2006). NLRP3, ASC, caspase-1, and IL-1β mRNA expression levels were normalized to the expression of the glyceraldehyde-3-phosphate dehydrogenase (GAPDH).

### 2.17 Western blotting

Tissue and cells were lysed with a protein extraction kit (#KGP250, KeyGen) and protein concentrations analyzed using the Bradford assay (#KGA801, KeyGen). Proteins were separated via electrophoresis, transferred to polyvinylidene difluoride membranes (Millipore, Darmstadt, Germany), blocked with 5% bovine serum albumin, and incubated with primary antibodies against p-NF-κB (1:2000; #AF2006, Affinity Biosciences), NF-κB (1:100; #AF5006, Affinity Biosciences), NLRP3 (1:2000; #DF7438, Affinity Biosciences), ASC (1:2000; #DF6304, Affinity Biosciences), cleaved caspase-1 (1:2000; #AF4022, Affinity Biosciences), caspase-1 (1:2000; #AF5418, Affinity Biosciences), IL-1β (1:2000; #AF5103, Affinity Biosciences), and GAPDH (1:2000; #BK7021, Bioker, Zhejiang, China) at 4°C for 12 h. Suitable secondary antibodies were applied at room temperature for 1 h, and protein bands visualized using chemiluminescence (ChemiDoc MP, Bio-Rad, California, United States).

### 2.18 Immunofluorescence staining

RAW264.7 macrophages were fixed with 4% paraformaldehyde, blocked with 5% bovine serum albumin, and incubated with primary antibodies against NLRP3, ASC, cleaved caspase-1, IL-1β, Gasdermin D (1:200; GSDMD, #AF4012, Affinity Biosciences), cluster of differentiation 86 (1:400; CD86, #A00220-4, Boster Biosciences, Hubei, China) and mannose receptor (1:400; CD206, #A02285-2, Boster Biosciences) at 4°C overnight. After washing with Tris-buffered saline, secondary goat anti-mouse lgG antibodies conjugated with an Alexa Fluor^®^ 488 (1:1000; #ab150113, Abcam, Cambridge, United Kingdom) were applied. The cells were then washed, mounted with mounting medium containing DAPI, and imaged under a confocal laser scanning microscope (#LSM800, Zeiss, Oberkochen, Germany). Images were analyzed using Zeiss software (MicroImaging GmbH, Zeiss).

### 2.19 Ultra-high performance liquid chromatography with quadrupole time-of-flight mass spectrometry (UPLC-QTOF-MS) analysis

A UPLC system (ACQUITY, Waters, Massachusetts, United States) coupled to a mass spectrometer (X-500R, AB SCIEX, California, United States) was used for UPLC-QTOF-MS analysis. Separation was performed on ACQUITY UPLC BEH C18 columns (2.1 × 150 mm, 1.7 μm; Waters). The injection volume was 5 μL. The mobile phase consisted of solvent A (acetonitrile, containing 0.1% formic acid) and solvent B (H_2_O, containing 0.1% formic acid). The gradient conditions were as follows: 0–6 min, 1%–10% A; 6–9 min, 10%–25% A; 9–20 min, 25%–45% A; 20–25 min, 45%–99% A. The flow rate was 0.30 mL/min. The MS conditions were set as follows: spray voltage of 5.5/−4.5 kV, collision energy of 35 ± 15 eV, and turbo spray temperature of 600°C. The mass-charge ratio (*m/z*) scan range was collected from 50 DA to 1500 DA in positive and negative modes.

### 2.20 Network analysis

The potential targets of the absorbed components of YNJ were acquired from the Comparative Toxicogenomics Database (https://www.ctdbase.org) and the Traditional Chinese Medicine Systems Pharmacology Database and Analysis Platform (http://tcmspw.com/tcmsp.php) (Ru et al., 2014). The Kyoto Encyclopedia of Genes and Genomes (KEGG) database (Minoru et al., 2023) was accessed to infer the pharmacodynamic mechanism of action of YNJ on potential molecular targets. The intersecting targets retrieved in RNA-seq and bioinformatics analysis were imported into the STRING database (https://string-db.org/cgi/input). The free nodes were used construct a protein‒protein interaction (PPI) network and further processed by Cytoscape 3.9.1 software for visualization, and the YNJ pharmacodynamic ingredients were ultimately obtained (Jin et al., 2016).

### 2.21 Molecular docking

The structures of the active YNJ ingredients were downloaded from PubChem (https://pubchem.ncbi.nlm.nih.gov/), while the 3D structures of the docking targets (NF-κB/NLRP3 pathway proteins) were downloaded from the Worldwide Protein Data Bank database (https://www.rcsb.org/) (Liu Y. Y. et al., 2021). Preprocessing was performed by importing data into AutoDock4.2 software (Center for Computational Structural Biology, California, United States), and docking results were visualized using PyMol software (Baugh et al., 2011).

### 2.22 Statistical analysis

Statistical analysis was performed using GraphPad Prism 9.0 (GraphPad, California, US). One-way ANOVA was used to determine statistical significance between groups. Data are shown as the mean ± standard deviation (SD). Statistical significance was set at *P* < 0.05.

## 3 Results

### 3.1 YNJ attenuates LPS-induced ALI in mice

**FIGURE 1:**
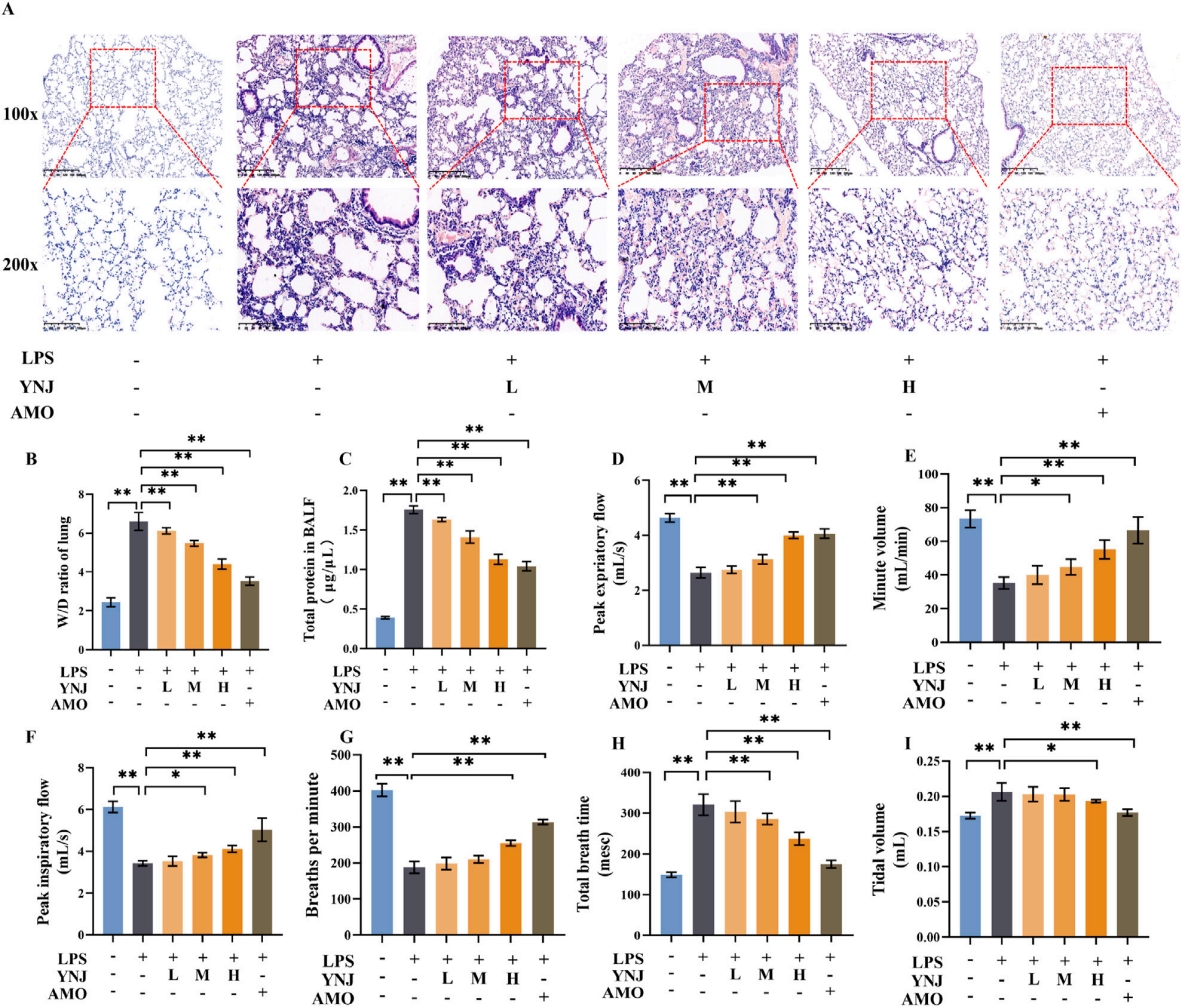
YNJ alleviates ALI and improves respiration in LPS-treated mice. **(A)** HE analysis. **(B)** Lung W/D ratio measurements. **(C)** Protein levels in BALF. **(D)** Peak expiratory flow. **(E)** Minute volume. **(F)** Peak inspiratory flow. **(G)** Breathing time per minute. **(H)** Total breathing time. **(I)** Tidal volume. The data are presented as the means ± SD (n = 5). *P < 0.05, **P < 0.01.

To evaluate the therapeutic effect of YNJ on ALI, we established an LPS-induced ALI mouse model and treated the mice with low (L), medium (M), and high dose (H) YNJ. HE analysis revealed that LPS treatment led to thickened lung septum, inflammation, and pulmonary interstitial edema (Figure 1A). In contrast, intact lung tissue structures with largely preserved septum thickness were observed in the YNJ-treated groups. The lung W/D ratio was assessed to evaluate pulmonary edema (Figure 1B). Compared with the control group, the W/D ratio of the lung was increased by LPS (*P* < 0.01). However, in LPS-treated mice, YNJ reduced the W/D ratio in a dose-dependent manner (*P* < 0.01). As expected, a reduced lung W/D ratio was also observed in ALI mice administered AMO (*P* < 0.01). The degree of pulmonary microvascular permeability was determined based on the protein concentrations measured in the BALF. Compared with the control group, the LPS-treated group exhibited increased BALF protein levels (*P* < 0.01). In contrast, the protein content in the BALF was decreased in ALI mice treated with YNJ (*P* < 0.01), which achieved, at the highest dose, similar efficacy to that of AMO (Figure 1C). These findings indicate that YNJ protects against LPS-induced ALI.

### 3.2 YNJ improves respiration in LPS-induced mice

We next examined the effect of YNJ on respiratory function in LPS-treated mice. Compared with the control group, in LPS-treated mice peak expiratory flow (Figure 1D), minute volume (Figure 1E), peak inspiratory flow (Figure 1F), and breathing rate (Figure 1G) were significantly decreased (*P* < 0.01 for all). In turn, increased total breath time (Figure 1H) and tidal volume (Figure 1I) were also observed after LPS exposure (*P* < 0.01 for both measures). Notably, YNJ treatment improved minute volume (*P* < 0.05 for YNJ-M, *P* < 0.01 for YNJ-H), peak expiratory flow (*P* < 0.01 for YNJ-M and YNJ-H), peak inspiratory flow (*P* < 0.05 for YNJ-M, *P* < 0.01 for YNJ-H), breathing rate (*P* < 0.01 for YNJ-H), total breath time (*P* < 0.01 for YNJ-M and YNJ-H), and total volume (*P* < 0.05 for YNJ-H). Comparable effects to those elicited by YNJ were observed in AMO-treated mice (*P* < 0.01). The above data indicate that YNJ improves respiration in LPS-treated mice.

### 3.3 YNJ inhibits pyroptosis in ALI mice

**FIGURE 2:**
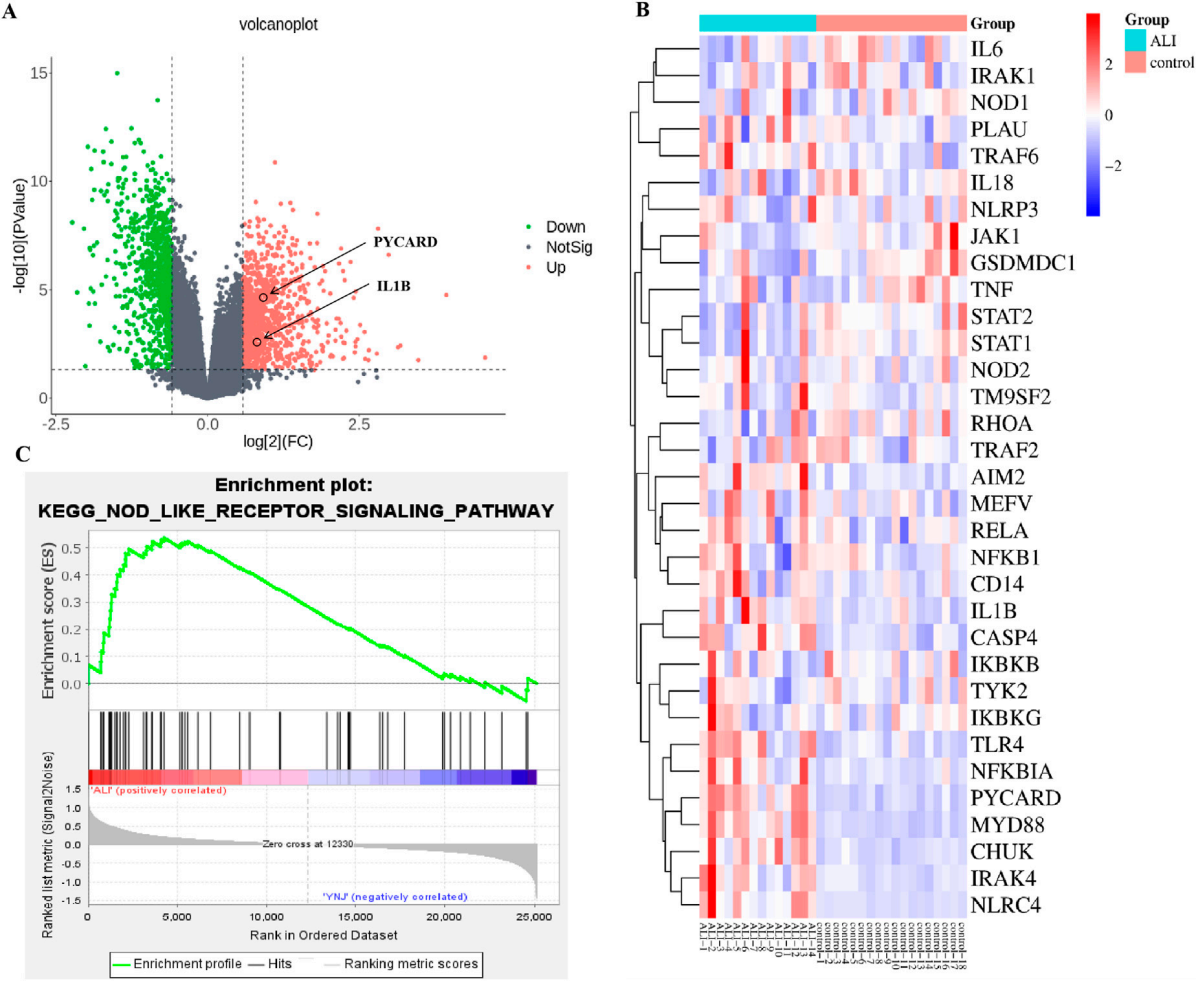
Analysis of RNA-seq profiles from pneumonia patients with lung injury and normal lung samples. **(A)** Volcano plot visualization of DEGs. **(B)** Heatmap of pyroptosis-related genes. **(C)** GSEA enrichment results.

To evaluate whether protection of lung integrity afforded by YNJ is related to inhibition of pyroptosis, we first examined clinical expression of pyroptosis-related genes in 14 severe pneumonia samples with lung injury and 18 normal lung samples contained in the GSE40012 dataset. Volcano plots (Figure 2A) revealed, among 36094 genes, 1791 DEGs, of which 1015 were upregulated and 776 were downregulated. Differential expression was noted for pyroptosis-related genes (Figure 2B), with IL1B showing marked upregulation in pneumonia compared to normal lung samples. DEG enrichment by gene set enrichment analysis (GSEA) suggested that ALI was closely related to the NOD-like receptor signaling pathway (Figure 2C).

**FIGURE 3:**
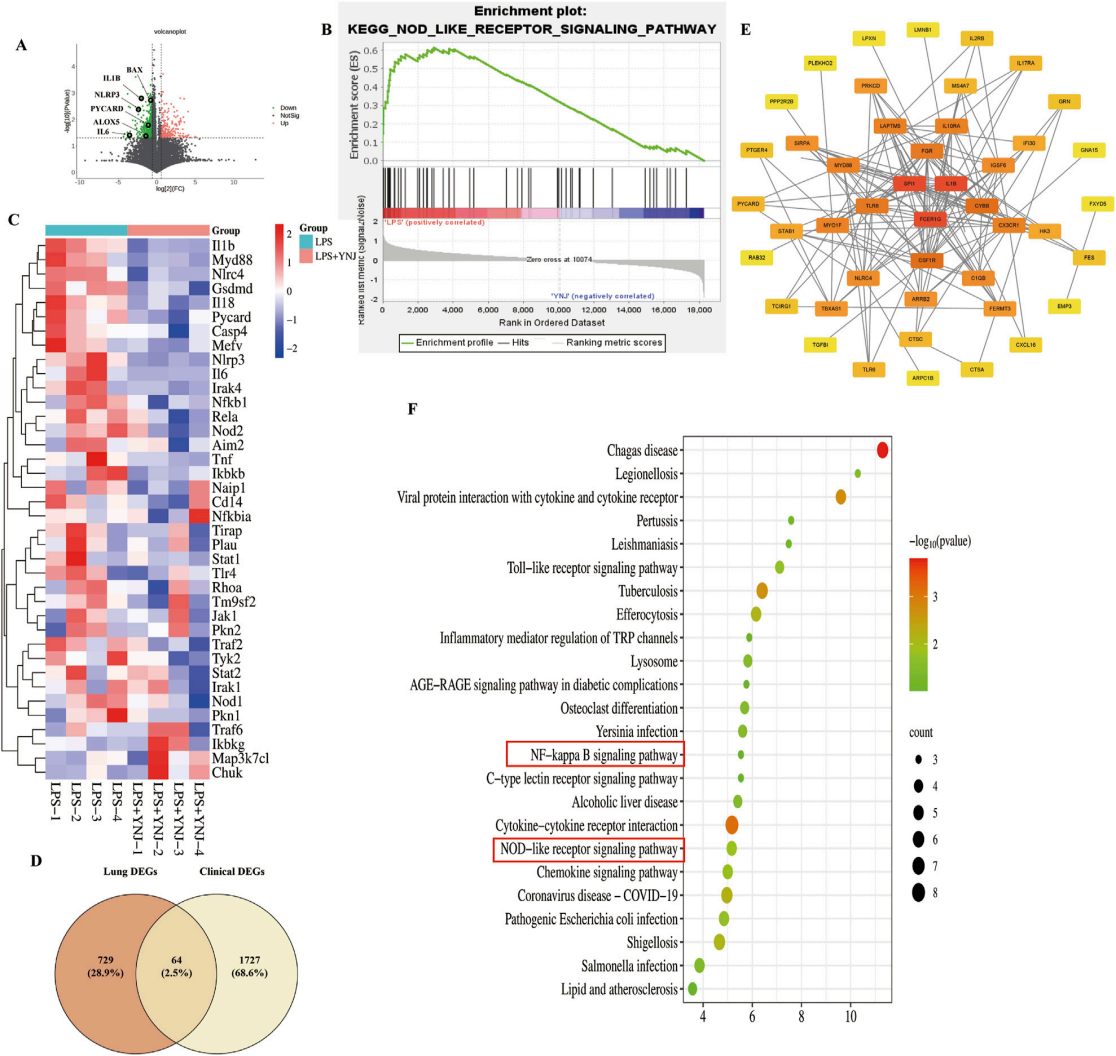
RNA-seq analysis of mouse lung samples. **(A)** Volcano plot of DEGs. **(B)** GSEA results. **(C)** Heatmap of pyroptosis-related genes **(D)** Venn analysis integrating DEGs retrieved from lung tissues from ALI mouse and GSE40012 pneumonia patients. **(E)** PPI network of the common DEGs. **(F)** KEGG enrichment analysis of commonly shared DEGs.

Furthermore, we performed RNA-seq analysis of mouse lung tissues to explore the mechanism of action of YNJ on ALI. A total of 793 DEGs were identified (Figure 3A). Among the lung injury-related genes (Supplementary Figure S1), the expression of IL-6, Bcl-2 associated X (BAX), and arachidonate 5-lipoxygenase (ALOX5) was decreased after YNJ treatment. Interestingly, GSEA indicated that YNJ may potentially regulate the NOD-like receptor signaling pathway (Figure 3B). Additionally, the expression of pyroptosis-related genes, such as NLRP3 and IL1B, was downregulated by YNJ (Figure 3C). A total of 64 common DEGs were found after comparing RNA-seq data from lungs of ALI mouse and pneumonia patients in the GSE40012 dataset (Figure 3D). A PPI network next was constructed according to these 64 common targets, which revealed SPL1, FCER1G, and IL1B as the three top DEGs (Figure 3E). KEGG enrichment analysis was also performed on the 64 common DEGs (Figure 3F), with results further indicating that the pyroptosis-related pathway is involved in the therapeutic effect of YNJ in ALI.

### 3.4 YNJ inhibits the NF-κB/NLRP3 pathway in the lungs of LPS-treated mice

**FIGURE 4:**
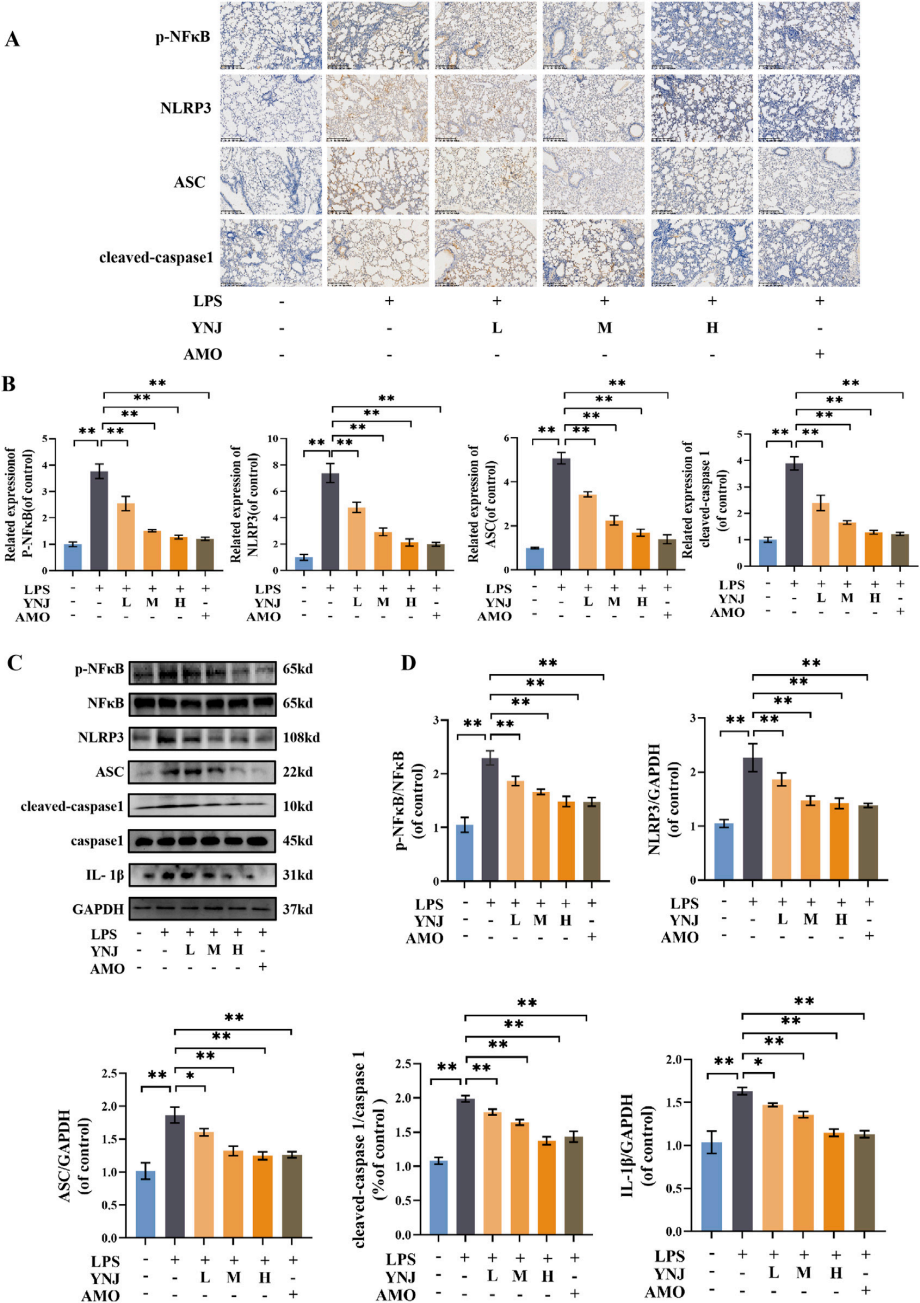
YNJ inhibits NF-κB/NLRP3-mediated pyroptosis in the lungs of LPS-treated mice. **(A, B)** Representative IHC images and corresponding statistical analysis (n = 5). **(C, D)** Representative Western blotting images and corresponding statistical analysis (n = 3). The data are presented as the mean ± SD. *P < 0.05, **P < 0.01.

To validate the involvement of the NF-κB/NLRP3 pathway in the protective effect of YNJ against ALI, the expression of p-NF-κB, NLRP3, ASC, and cleaved caspase-1 in mouse lung tissue was examined by IHC. Results showed that compared with the control group, LPS increased p-NF-κB, NLRP3, ASC, and cleaved caspase-1 levels (*P* < 0.01 for all), while YNJ and AMO reversed these changes (*P* < 0.01) (Figures 4A, B). Western blotting confirmed that LPS increased the expression of p-NF-κB and total NF-κB, NLRP3, ASC, cleaved-caspase1 and total caspase1 (*P* < 0.01 for all), and IL-1β (*P* < 0.05 for YNJ-L, *P* < 0.01 for YNJ-H) in lung tissue, whereas YNJ and AMO treatments decreased the expression of these proteins (*P* < 0.05 for YNJ-L on ASC and IL-1β, *P* < 0.01 for others). These results suggest that YNJ exerts therapeutic effects in ALI through inhibiting NF-κB/NLRP3-mediated pyroptosis in lung tissue.

### 3.5 YNJ protects MLE-12 cells against LPS-induced injury

**FIGURE 5:**
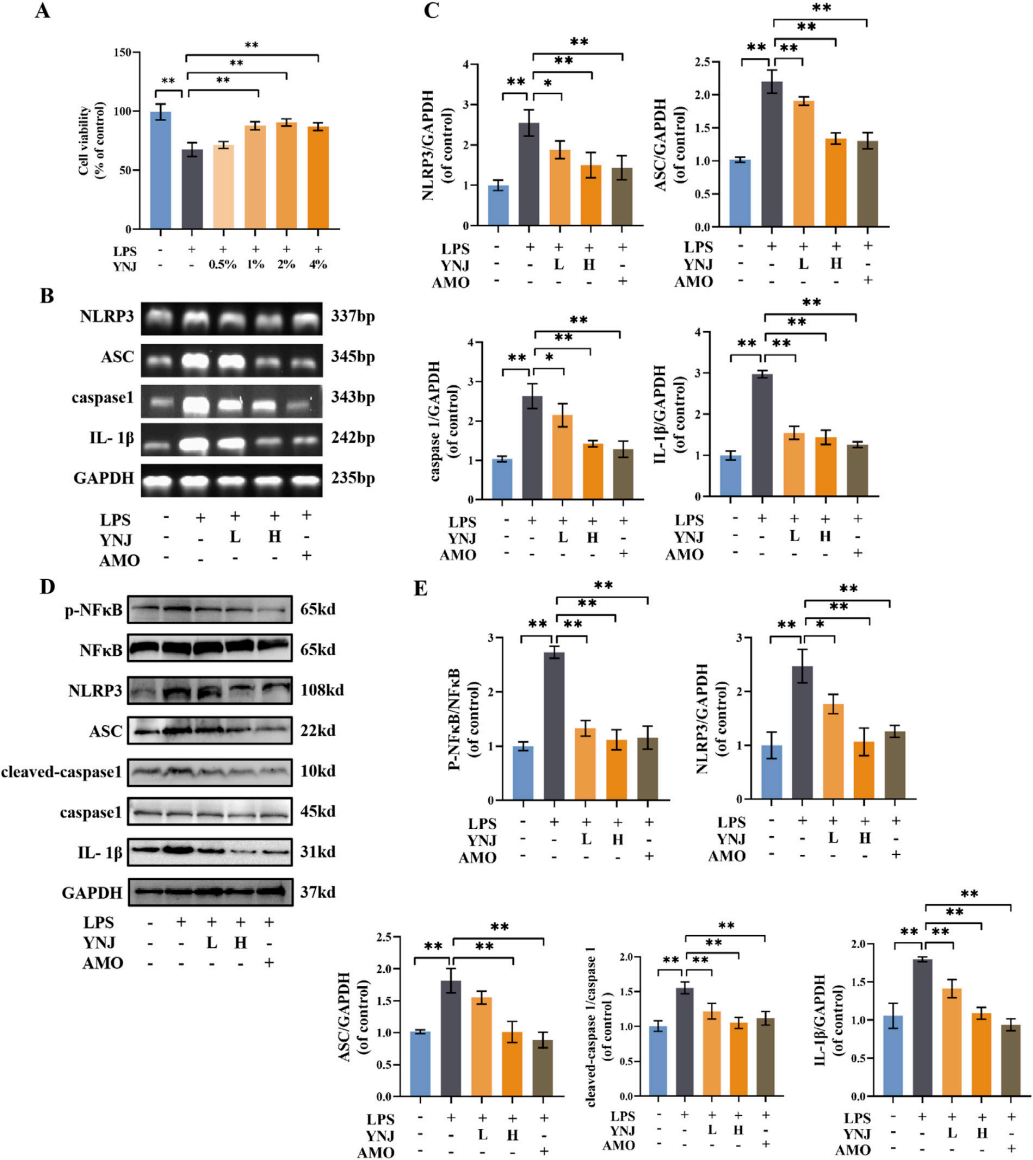
YNJ suppresses LPS-induced pyroptosis in MLE-12 cells by inhibiting the NF-κB/NLRP3 pathway. **(A)** Cell viability assay results (n = 6). **(B, C)** Images of agarose gel electrophoresis for PCR products and corresponding statistical analysis (n = 3). **(D, E)** Western blot images and corresponding statistical analysis (n = 3). The data are presented as the mean ± SD. *P < 0.05, **P < 0.01.

To study the effect of YNJ on lung epithelial cells, LPS-exposed mouse alveolar MLE-12 cells were treated with serum from mice treated or not with YNJ. Compared to untreated control cells, viability was decreased in those treated with LPS (*P* < 0.01), and this effect was counteracted upon exposure to YNJ-containing serum (*P* < 0.01) (Figure 5A). LPS induced also oxidative stress in MLE-12 cells, as evidenced by increased MDA and ROS levels, and these changes were effectively reversed by YNJ-containing serum treatment (Supplementary Figure S2). The above data indicates that YNJ can protect MLE-12 cells against LPS-induced oxidative damage.

### 3.6 YNJ suppresses pyroptosis by inhibiting the NF-κB/NLRP3 pathway in LPS-treated lung epithelial cells

To investigate whether YNJ protects lung epithelial cells from LPS-induced death by inhibiting pyroptosis, PCR analysis was conducted in cultured MLE-12 cells (Supplementary Table S1). Results showed that LPS exposure upregulated IL-1β, ASC, caspase1, and NLRP3 mRNA expression (*P* < 0.01 for all), while simultaneous exposure of YNJ-containing serum or AMO downregulated the expression of these genes (Figures 5B, C). These findings were reproduced by Western blot analyses, which revealed that YNJ and AMO decreased p-NF-κB and total NF-κB, NLPR3, ASC, cleaved-caspase1 and total caspase1, and IL-1β protein levels (Figures 5D, E). These results indicated that YNJ inhibits LPS-induced pyroptosis in lung epithelial cells by suppressing the NF-κB/NLRP3 pathway.

### 3.7 YNJ promotes M2 polarization in LPS-stimulated macrophages

**FIGURE 6:**
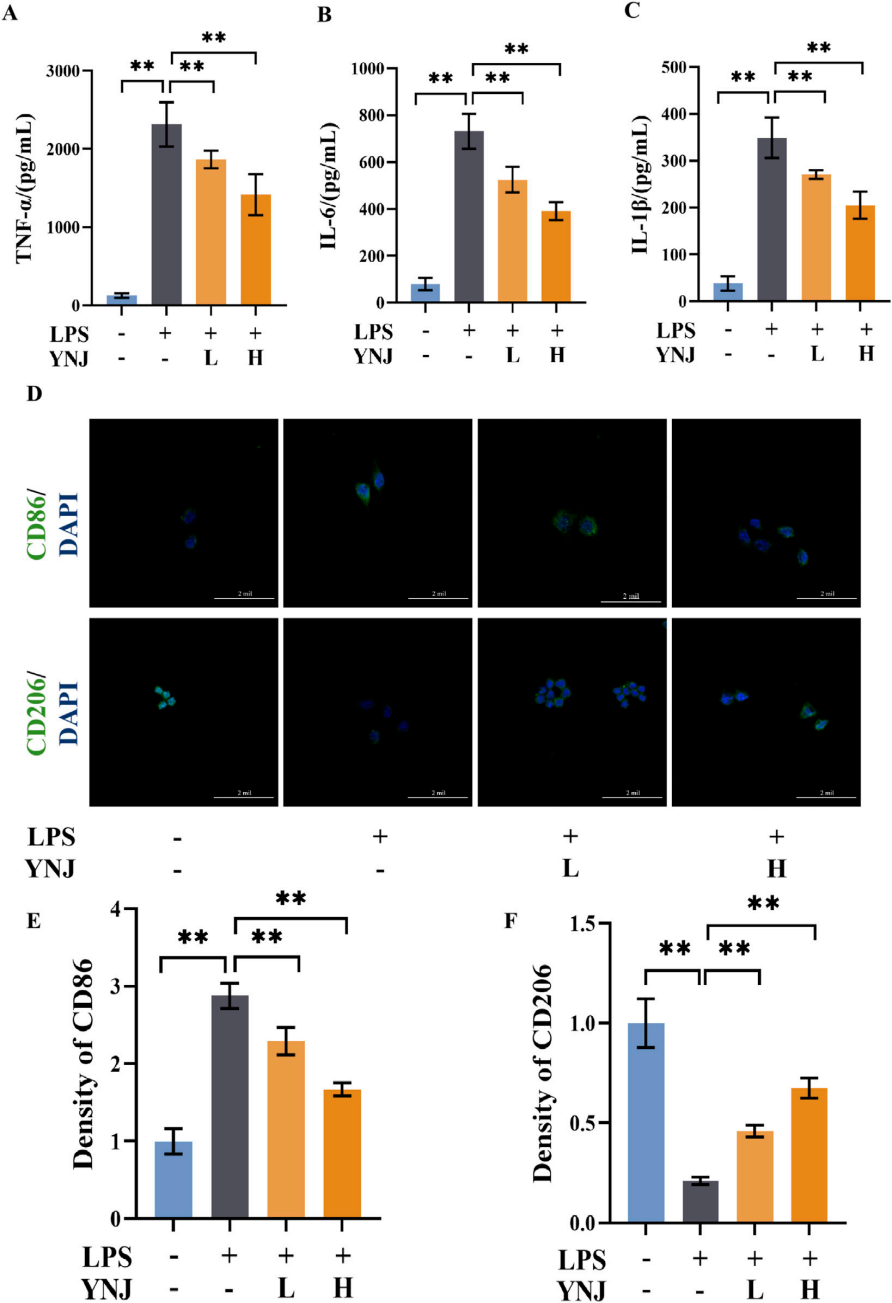
YNJ reduces the secretion of inflammatory factors and promotes M2 macrophage polarization. **(A–C)** ELISA of TNF-α **(A)**, IL-6 **(B)**, and IL-1β **(C)** contents in the supernatant of cultured RAW264.7 macrophages. **(D)** IF images of CD86 and CD206 detection in cultured RAW264.7 macrophages. **(E, F)** Statistical analysis of CD86 and CD206 expression by IF. The data are presented as the mean ± SD (n = 6). **P < 0.01.

We next evaluated the effect of YNJ on the polarization status of RAW264.7 macrophages. Compared with the control group, LPS stimulated the secretion of the proinflammatory cytokines TNF-α, IL-6, and IL-1β (*P* < 0.01 for all), while concomitant treatment with YNJ-containing serum attenuated these changes (Figures 6A–C). Immunofluorescence (IF) detection of the M1-type macrophage marker protein CD86 and M2-type macrophage marker protein CD206 (Figure 6D) showed that LPS increased the expression of CD86 (*P* < 0.01; Figure 6E), indicating polarization of macrophages toward the M1-type. In contrast, after YNJ exposure, increased expression of CD206 (*P* < 0.01; Figure 6F) was noted in LPS-treated macrophages. The above results indicated that YNJ promotes M2 polarizationof macrophages.

### 3.8 YNJ suppresses pyroptosis by inhibiting NF-κB/NLRP3 signaling in LPS-treated macrophages

**FIGURE 7:**
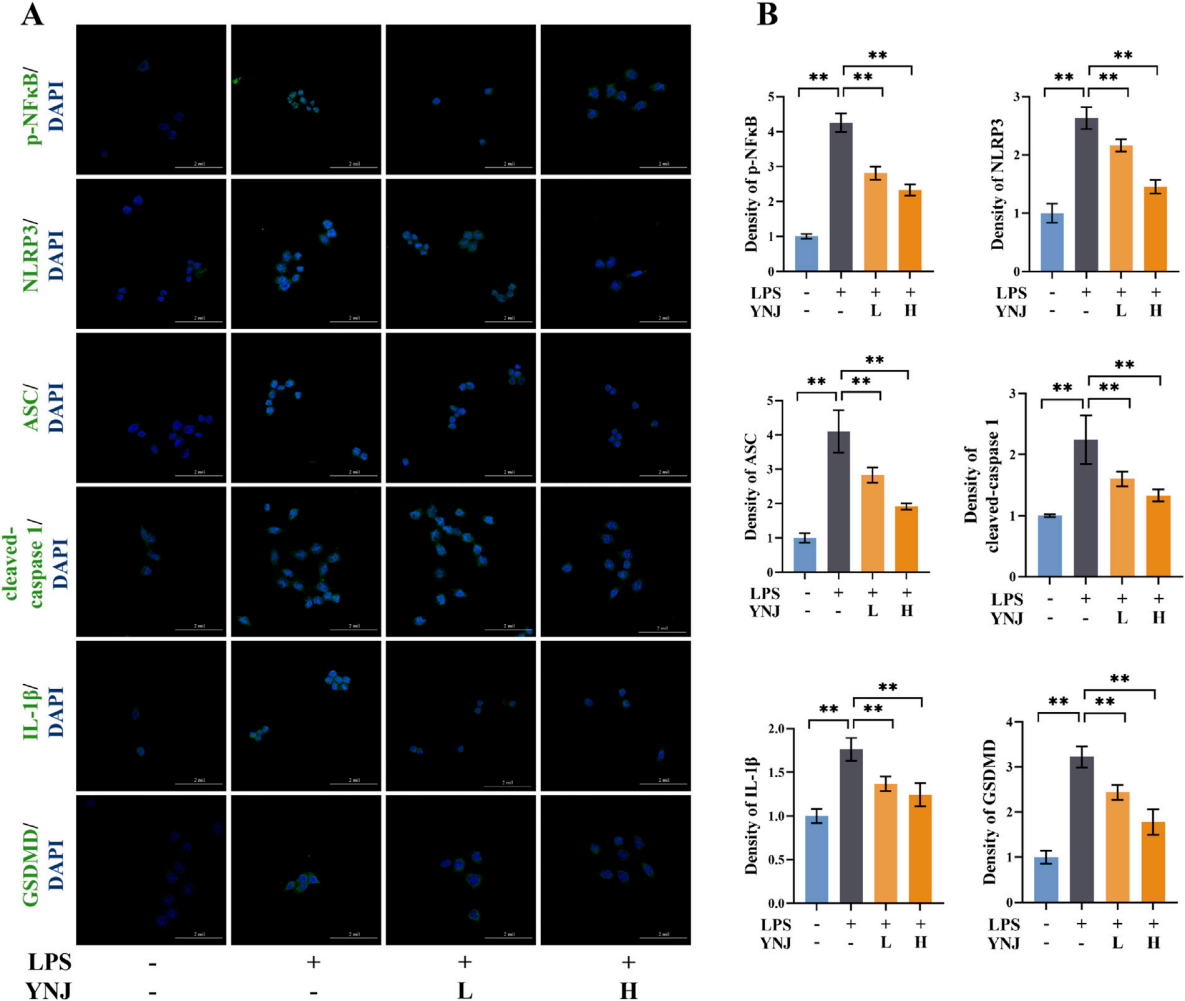
YNJ suppresses pyroptosis in LPS-induced macrophages by inhibiting the NF-κB/NLRP3 pathway. **(A, B)** IF images of p-NF-κB, NLRP3, ASC, cleaved caspase-1, IL-1β, and GSDMD detection and corresponding statistical analysis (n = 6). The data are presented as the mean ± SD. **P < 0.01.

Pyroptosis-related proteins were next detected by IF in LPS-stimulated macrophages. Results showed that LPS upregulated the expression of p-NF-κB, NLRP3, ASC, cleaved caspase-1, IL-1β, and GSDMD, while concomitant exposure to YNJ-containing serum reversed these changes (*P* < 0.01; Figures 7A, B). These results suggest that YNJ inhibits the NF-κB/NLRP3 pathway in LPS-induced macrophages.

### 3.9 NLRP3 activation abolishes the pro-survival effect of YNJ on MLE-12 and RAW264.7 cells

The NLRP3 pathway agonist nigericin and LPS/ATP were used to verify the effect of YNJ on the NLRP3 pathway. Compared with the YNJ group, nigericin addition reduced the viability of MLE-12 cells (Supplementary Figure S3). In turn, cell viability was decreased by LPS/ATP treatment (Supplementary Figure S4), and YNJ addition reversed this change. Meanwhile, in LPS-treated macrophages, viability was reduced in the YNJ + nigericin group compared to the YNJ group (Supplementary Figure S5), and increased instead in LPS/ATP-treated macrophages co-treated with YNJ (Supplementary Figure S6). These data confirmed that YNJ promotes survival of lung epithelial cells and macrophages by inhibiting NLRP3 targets.

### 3.10 Serum pharmacochemistry analysis

**FIGURE 8:**
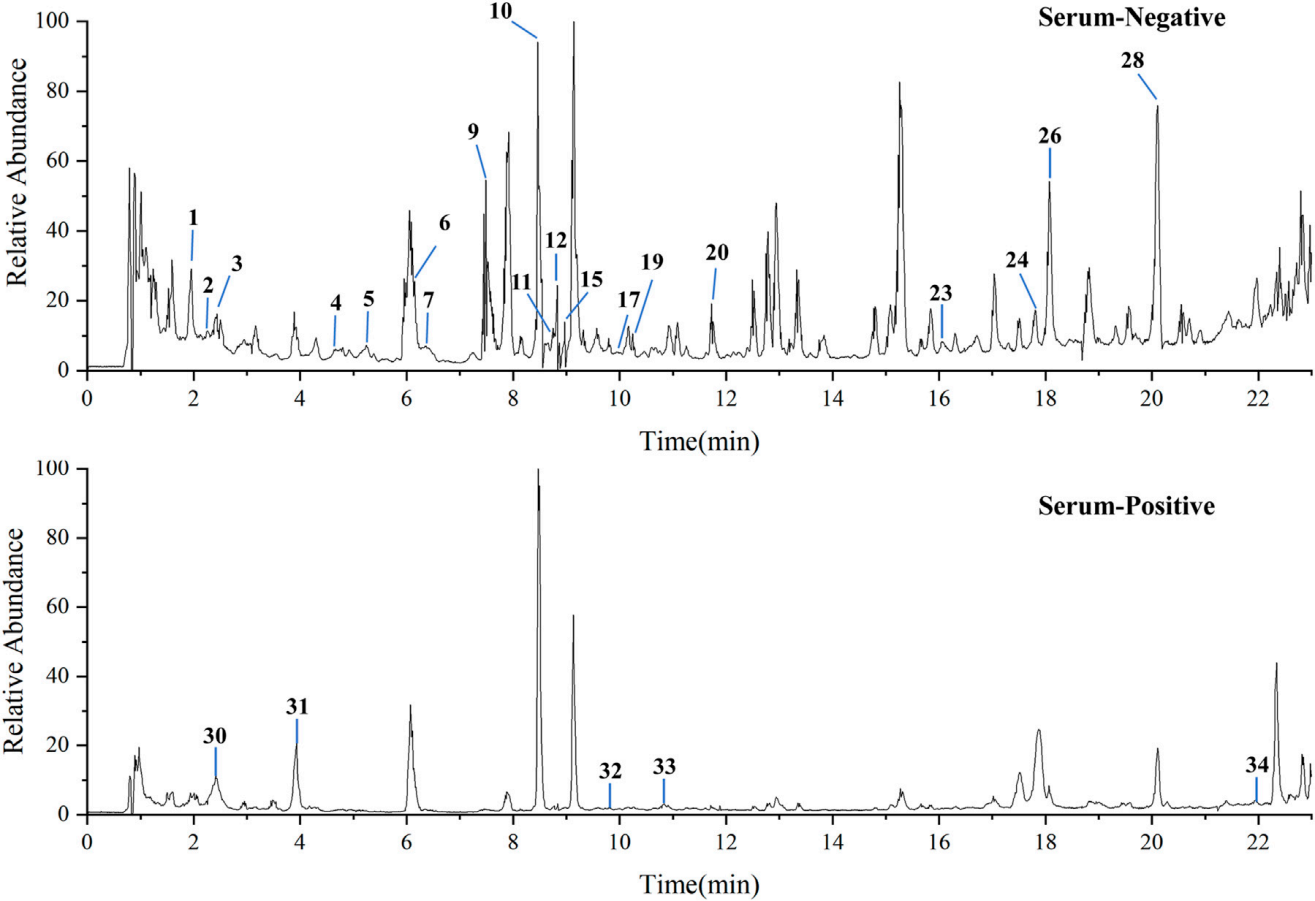
Total ion chromatograms of YNJ drug-containing serum in the positive-ion and negative-ion modes.

UPLC-QTOF-MS analysis of YNJ water extract (Supplementary Figure S7) and YNJ-containing serum were performed to explore the active ingredients of YNJ (Figure 8). A total of 34 compounds were identified in the YNJ water extract, while 23 prototype compounds were found in the YNJ drug-containing serum. There were 2 phenylpropanes, 4 iridoid terpenes, 2 triterpenoid saponins, 3 flavonoids, 5 organic acids, 5 steroid saponins, and other constituents (Supplementary Table S2).

### 3.11 Interactions between the absorbed ingredients of YNJ and the NF-κB/NLRP3 pathway

Network analysis and molecular docking were applied to study the interactions between the absorbed components of YNJ and members of the NF-κB/NLRP3 pathway. A total of 518 targets were associated with the 23 absorbed YNJ ingredients. Of these, seven ingredients were related with seven targets among the common DEGs identified by comparison of RNA-seq data between ALI mouse lung tissue and pneumonia patients’ lung samples in the GSE40012 dataset (Supplementary Figure S8A). Furthermore, docking simulations predicted that four components from YNJ, namely, diosgenin, timosaponin BII, caffeic acid, and mangiferin, could bind to IL-1β (Supplementary Figure S8B). These data further suggested that regulation of the NF-κB/NLRP3 pathway contributes to the therapeutic effect of YNJ on ALI (Supplementary Figure S9).

### 3.12 Pharmacodynamic verification of YNJ absorption ingredients

To verify the anti-inflammatory activities of the predicted ingredients, MLE-12 and macrophages were treated with diosgenin, timosaponin BII, caffeic acid, and mangiferin at different concentrations. The four components effectively reversed the cell viabilities of the LPS-induced MLE-12 (Supplementary Figure S10) and macrophages (Supplementary Figure S11). Their effects on the polarization of macrophages were detected by immunofluorescence (Supplementary Figure S12A). Among them, diosgenin, timosaponin BII, and mangiferin treatments decreased the expression of CD86 (Supplementary Figure S12B) and increased the expression of CD206 in LPS-treated macrophages (Supplementary Figure S12C). These results indicated that diosgenin, timosaponin BII, and mangiferin exerted anti-inflammatory effects in the LPS-induced MLE-12 and macrophages.

## 4 Discussion

Although the pathological mechanisms of ALI have been extensively studied, there is still in clinical practice a lack of specific treatment drugs (Liu et al., 2022). The present study revealed that pyroptosis plays an essential role in ALI. YNJ is a classic and widely-used traditional Chinese herbal formula first reported about 400 years ago (Lv et al., 2024) and is commonly used in the clinical treatment of inflammatory diseases. Our finding that YNJ attenuated pyroptosis in the lungs of LPS-treated mice, as well as in alveolar epithelial cells and macrophages *in vitro*, suggested that YNJ may be effective in relieving ALI severity. Importantly, we showed that negative regulation of the NF-κB/NLRP3 pathway is part of the therapeutic effects of YNJ, and demonstrated specific inhibitory activity on NF-κB/NLRP3-mediated pyroptosis for three absorbed constituents of YNJ.

ALI is characterized by acute pulmonary dysfunction, hypoxic cyanosis, reduced lung compliance, and diffuse alveolar infiltration (Mokrá, 2020). LPS, a component of the outer wall of Gram-negative bacteria, has been shown to be a potent inducer of cellular inflammation that can cause damage to multiple organs (Guo et al., 2015; Wang L. et al., 2022). Our previous work demonstrated the efficacy of YNJ in treating LPS-induced periodontitis (Ye et al., 2024). In this study, LPS was used to establish an *in vivo* model and two *in vitro* models of ALI (i.e., alveolar epithelial MLE-12 cells and RAW264.7 macrophages). Consistent with previously reported results (Tang et al., 2021; Chen et al., 2023), LPS not only disrupted the pulmonary morphology and respiratory function of mice but also suppressed the viability of alveolar epithelial cells. Furthermore, LPS exposure induced M1 polarization of macrophages and triggered the associated inflammatory cascade. In clinical practice, conventional treatment methods such as lung-protective ventilation and neuromuscular blockers are limited due to their side effects (Lewis et al., 2019; Nanchal and Truwit, 2018). Our preclinical work reveals a robust therapeutic effect of YNJ and offers new insight into drug development for ALI treatment.

**FIGURE 9:**
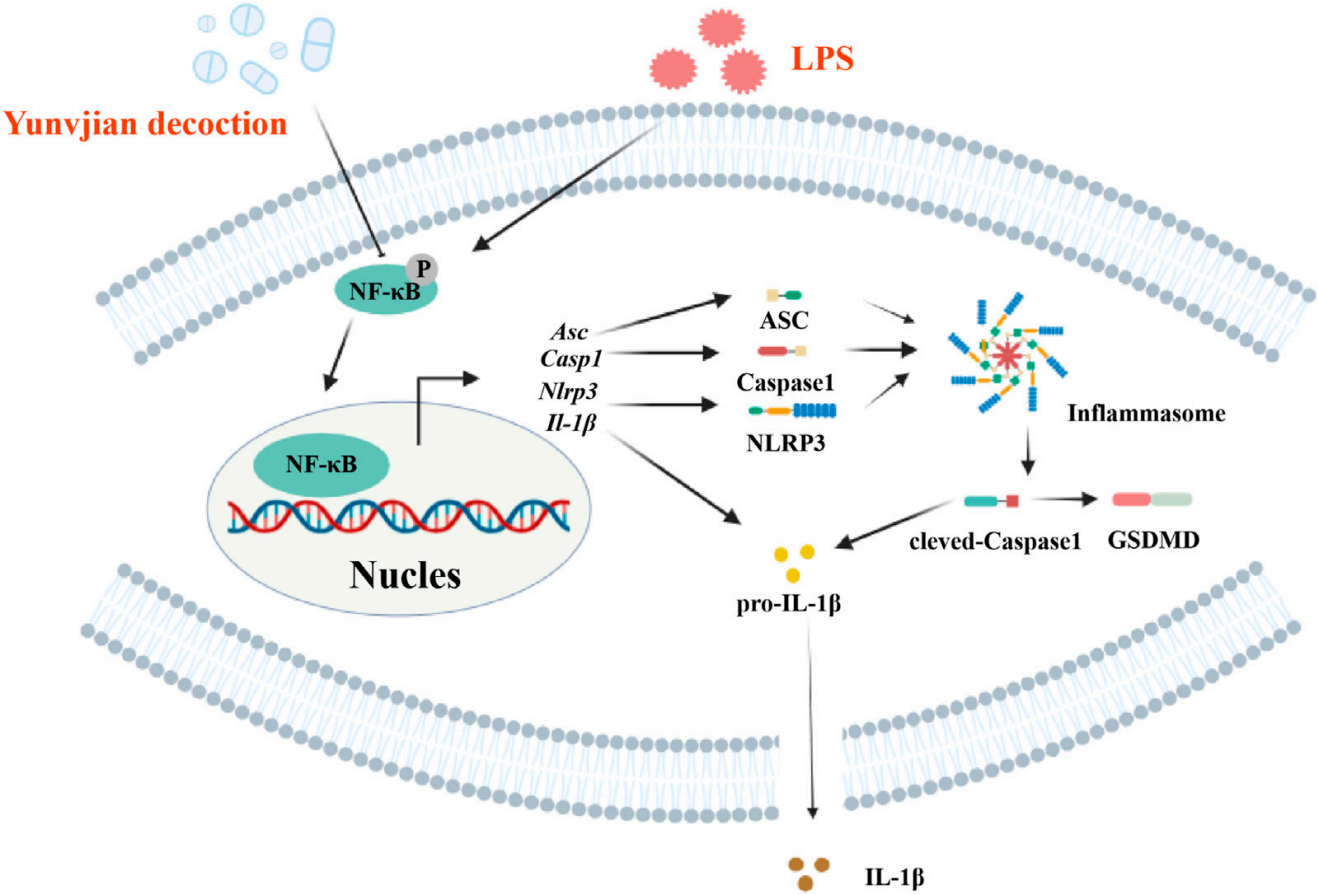
Schematic diagram of the proposed therapeutic mechanism of YNJ in ALI.

The NLRP3 inflammasome, a vital component of innate immunity, promotes the secretion of IL-1β via caspase-1 activation, thereby inducing pyroptosis (Wang and Hauenstein, 2020; Shi et al., 2015; Liu et al., 2016). NF-κB is involved in the activation of NLRP3 (Dapueto et al., 2021), and the NF-κB/NLRP3 pathway is a critical mechanism in ALI pathogenesis (Hong et al., 2022). Activation of the NLRP3 inflammasome has been shown to exacerbate inflammation in ALI by mediating macrophage pyroptosis (Zhang et al., 2016). In this work, we used RNA-seq combined with bioinformatics analysis to explore the potential mechanism of action of YNJ in the treatment of ALI, and identified 64 common DEGs between lung tissues from ALI mouse and patients with pneumonia-related lung injury. Subsequent KEGG analysis further identified the NF-κB/NLRP3 pathway as a potential target of YNJ. Our *in vivo* and *in vitro* results proved that YNJ exerted an anti-pyroptotic effect by inhibiting NF-κB and NLRP3 in LPS-exposed lung, MLE-12 cells, and macrophages. These results suggest that YNJ can alleviate LPS-induced ALI symptoms by inhibiting alveolar epithelial pyroptosis via the NF-κB/NLRP3 pathway (Figure 9).

Furthermore, we identified a total of 23 bioactive YNJ components by serum pharmacochemistry, and used network analysis to analyze their potential regulatory effects on NF-κB/NLRP3 pathway proteins. Results suggested that four YNJ bioactive ingredients, namely, diosgenin from *Achyranthes bidentata Blume*, timosaponin BII and mangiferin from *Anemarrhena asphodeloides* Bunge., and caffeic acid from *Rehmannia glutinosa* (Gaertn.) DC., have potential regulatory effects on the NF-κB/NLRP3 pathway. Previous research showed that mangiferin reduced the cleavage of GSDMD in LPS-induced bone marrow-derived macrophages by inhibiting the NF-κB pathway and the pro-inflammatory caspase-mediated pyroptosis cascade (Feng M. et al., 2022). Timosaponin BII was reported to inhibit NF-κB and reduce the expression of proinflammatory cytokines in microglial cells (Lu et al., 2009). Caffeic acid was found to protect macrophages from LPS-induced noncanonical pyroptosis and alleviate LPS-induced sepsis in mice (Liu et al., 2023), and ameliorated iron-induced tissue damage through potential inhibition of carbonic anhydrase (Figueredo et al., 2024). Subsequently, we verified the effectiveness of the four components, and the results showed that diosgenin, timosaponin BII and mangiferin all showed the effect of promoting the polarization of M2 macrophages. ALOX5 is an important target of proinflammatory factor secretion and oxidative stress (Šerý et al., 2016). Although caffeic acid did not show a direct effect on macrophages, in this work, ALOX5 acts as an important factor that can be regulated by YNJ, and is closely related to the role of caffeic acid. Our study thus revealed a potential regulatory role for several YNJ components on the NF-κB/NLRP3 pathway. Further investigation is necessary to evaluate the effects of the above YNJ components on ALI, as to reaffirm their therapeutic potential for clinical use.

## 5 Conclusion

Our findings demonstrate that YNJ alleviates ALI and improves lung function in LPS-treated mice. YNJ inhibited pyroptosis in lung tissue from ALI mice by inhibiting the NF-κB/NLRP3 pathway. Seven components of YNJ were identified as potential regulators of the NF-κB/NLRP3 pathway, with three among those promoting, under sepsis-mimicking conditions, lung alveolar epithelial cell survival and M2 polarization of macrophages. Further studies are planned to clarify additional molecular pathways affected by YNJ in ALI.

## Data Availability

The datasets presented in this study can be found in online repositories. The names of the repository/repositories and accession number(s) can be found in the article/Supplementary Material.

## Abbreviations

ALI, Acute lung injury; ALOX5, Arachidonate 5-lipoxygenase; AMO, Amoxicillin; ASC, Apoptosis-associated speck-like protein containing a CARD; ATP, Adenosine Triphosphate; BAX, Bcl-2 associated X; BALF, Bronchoalveolar lavage fluid; CD86, Cluster of Differentiation 86; CD206, Mannose receptor; DEGs, Differentially expressed genes; GAPDH, Glyceraldehyde-3-phosphate dehydrogenase; GSDMD, Gasdermin D; GEO, Gene Expression Omnibus; GSEA, Gene set enrichment analysis; HE, Hematoxylin-eosin; IHC, Immunohistochemical; IL-6, Interleukin-6; IL-1β, Interleukin-1β; KEGG, Kyoto Encyclopedia of Genes and Genomes; LPS, Lipopolysaccharide; MDA, Malondialdehyde; MLE-12, Murine lung epithelial cells-12; MTT, 3-(4,5-dimethylthiazol-2-yl)−2,5-diphenyltetrazolium bromide; NLRP3, NOD-like receptor protein 3; NF-κB, Nuclear factor-kappa B; PPI, Protein‒protein interaction; TNF-α, Tumor necrosis factor-α; UPLC-QTOF-MS, Ultra-high performance liquid chromatography with quadrupole time-of-flight mass spectrometry; W/D, Wet/dry weight; YNJ, Yunvjian decoction.

## References

[B1] Aziz M. Ode Y. Zhou M. Ochani M. Holodick N. E. Rothstein T. L. (2018). B-1a cells protect mice from sepsis-induced acute lung injury. Mol. Med. 24 (1), 26. 10.1186/s10020-018-0029-2 30134811 PMC6016888

[B2] Baugh E. H. Lyskov S. Weitzner B. D. Gray J. J. (2011). Real-time PyMOL visualization for rosetta and PyRosetta. PLoS One 6 (8), e21931. 10.1371/journal.pone.0021931 21857909 PMC3156697

[B3] Cao J. Li L. Yao Y. Xing Y. Ma H. (2022). Dehydroepiandrosterone exacerbates nigericin-induced abnormal autophagy and pyroptosis via GPER activation in LPS-primed macrophages. Cell Death Dis. 13, 372. 10.1038/s41419-022-04841-6 35440074 PMC9018772

[B4] Chen Z. Li J. Peng H. Zhang M. Wu X. Gui F. (2023). Meteorin-like/Meteorin-β protects LPS-induced acute lung injury by activating SIRT1-P53-SLC7A11 mediated ferroptosis pathway. Mol. Med. 29 (1), 144. 10.1186/s10020-023-00714-6 37880599 PMC10601160

[B5] Cheng P. Y. Li S. Y. Chen H. Y. (2021). Macrophages in lung injury, repair, and fibrosis. Cells 10 (2), 436. 10.3390/cells10020436 33670759 PMC7923175

[B6] Danielski L. G. Giustina A. D. Bonfante S. Barichello T. Petronilho F. (2020). The NLRP3 inflammasome and its role in sepsis development. Inflammation 43 (1), 24–31. 10.1007/s10753-019-01124-9 31741197

[B7] Dapueto R. Rodriguez-Duarte J. Galliussi G. Kamaid A. Bresque M. Batthyány C. (2021). A novel nitroalkene vitamin E analogue inhibits the NLRP3 inflammasome and protects against inflammation and glucose intolerance triggered by obesity. Redox Biol. 39, 101833. 10.1016/j.redox.2020.101833 33352465 PMC7750735

[B8] Du B. Cao L. Wang K. Miu J. Yao L. Xu Z. (2020). Peiminine attenuates acute lung injury induced by LPS through inhibiting lipid rafts formation. Inflammation 43 (3), 1110–1119. 10.1007/s10753-020-01198-w 32152924

[B9] Ehrentraut H. Weisheit C. K. Frede S. Hilbert T. (2019). Inducing acute lung injury in mice by direct intratracheal lipopolysaccharide instillation. J. Vis. Exp. 6–149. 10.3791/59999 31329180

[B10] Feng M. Wei S. Zhang S. Yang Y. (2022b). Anti-inflammation and anti-pyroptosis activities of mangiferin via suppressing NF-κB/NLRP3/GSDMD signaling cascades. Int. J. Mol. Sci. 23 (17), 10124. 10.3390/ijms231710124 36077522 PMC9456045

[B11] Feng Y. Li M. Yangzhong X. Zhang X. Zu A. Hou Y. (2022a). Pyroptosis in inflammation-related respiratory disease. J. Physiol. Biochem. 78 (4), 721–737. 10.1007/s13105-022-00909-1 35819638 PMC9684248

[B12] Feng Z. Qi S. Zhang Y. Qi Z. Yan L. Zhou J. (2017). Ly6G+ neutrophil-derived miR-223 inhibits the NLRP3 inflammasome in mitochondrial DAMP-induced acute lung injury. Cell Death Dis. 8 (11), e3170. 10.1038/cddis.2017.549 29144508 PMC5775410

[B13] Figueredo K. C. Guex C. G. Graiczik J. Reginato F. Z. Engelmann A. M. Andrade C. M. (2024). Caffeic acid and ferulic acid can improve toxicological damage caused by iron overload mediated by carbonic anhydrase inhibition. Drug Chem. Toxicol. 47 (2), 147–155. 10.1080/01480545.2022.2152043 36444844

[B14] Freeman T. L. Swartz T. H. (2020). Targeting the NLRP3 inflammasome in severe COVID19. Front. Immunol. 11, 1518. 10.3389/fimmu.2020.01518 32655582 PMC7324760

[B15] Georas S. N. Rezaee F. (2014). Epithelial barrier function: at the front line of asthma immunology and allergic airway inflammation. J. Allergy Clin. Immunol. 134 (3), 509–520. 10.1016/j.jaci.2014.05.049 25085341 PMC4170838

[B16] Guo L. Li S. Zhao Y. Qian P. Ji F. Qian L. (2015). Silencing angiopoietin-like protein 4 (ANGPTL4) protects against lipopolysaccharide-induced acute lung injury via regulating SIRT1/NF-kB pathway. J. Cell Physiol. 230 (10), 2390–2402. 10.1002/jcp.24969 25727991

[B17] Hashimoto M. Asai A. Kawagishi H. Mikawa R. Iwashita Y. Kanayama K. (2016). Elimination of p19ARF-expressing cells enhances pulmonary function in mice. JCI Insight 1 (12), e87732. 10.1172/jci.insight.87732 27699227 PMC5033852

[B18] Hong H. Lou S. Zheng F. Gao H. Wang N. Tian S. (2022). Hydnocarpin D attenuates lipopolysaccharide-induced acute lung injury via MAPK/NF-κB and Keap1/Nrf2/HO-1 pathway. Phytomedicine 101, 154143. 10.1016/j.phymed.2022.154143 35537248

[B19] Hung L. Y. Sen D. Oniskey T. K. Katzen J. Cohen N. A. Vaughan A. E. (2019). Macrophages promote epithelial proliferation following infectious and non-infectious lung injury through a Trefoil factor 2-dependent mechanism. Mucosal Immunol. 12 (1), 64–76. 10.1038/s41385-018-0096-2 30337651 PMC6301101

[B20] Jiang L. Zhang L. Kang K. Fei G. Gong R. Cao Y. (2016). Resveratrol ameliorates LPS-induced acute lung injury via NLRP3 inflammasome modulation. Biomed. Pharmacother. 84, 130–138. 10.1016/j.biopha.2016.09.020 27643555

[B21] Jin F. Ni X. Yu S. Jiang X. Zhou J. Mao D. (2016). Network pharmacology-based investigation of potential targets of triptonodiol acting on non-small-cell lung cancer. Eur. J. Med. Res. 28, 547. 10.1186/s40001-023-01453-4 PMC10683219 38017514

[B22] Jing W. Chunhua M. Shumin W. (2015). Effects of acteoside on lipopolysaccharide-induced inflammation in acute lung injury via regulation of NF-κB pathway *in vivo* and *in vitro* . Toxicol. Appl. Pharmacol. 285 (2), 128–135. 10.1016/j.taap.2015.04.004 25902336

[B23] Lee H. Abston E. Zhang D. Rai A. Jin Y. (2018). Extracellular vesicle: an emerging mediator of intercellular crosstalk in lung inflammation and injury. Front. Immunol. 9, 924. 10.3389/fimmu.2018.00924 29780385 PMC5946167

[B24] Lewis S. R. Pritchard M. W. Thomas C. M. Smith A. F. (2019). Pharmacological agents for adults with acute respiratory distress syndrome. Cochrane Database Syst. Rev. 7 (7), Cd004477. 10.1002/14651858.CD004477.pub3 31334568 PMC6646953

[B25] Li Y. Huang J. Foley N. M. Xu Y. Li Y. P. Pan J. (2016). B7H3 ameliorates LPS-induced acute lung injury via attenuation of neutrophil migration and infiltration. Sci. Rep. 6, 31284. 10.1038/srep31284 27515382 PMC4981866

[B26] Liu B. He R. Zhang L. Hao B. Jiang W. Wang W. (2021a). Inflammatory caspases drive pyroptosis in acute lung injury. Front. Pharmacol. 12, 631256. 10.3389/fphar.2021.631256 33613295 PMC7892432

[B27] Liu C. Xiao K. Xie L. (2022). Advances in the use of exosomes for the treatment of ALI/ARDS. Front. Immunol. 13, 971189. 10.3389/fimmu.2022.971189 36016948 PMC9396740

[B28] Liu M. Liu D. Yu C. Fan H. H. Zhao X. Wang H. (2023). Caffeic acid, but not ferulic acid, inhibits macrophage pyroptosis by directly blocking gasdermin D activation. MedComm (2020) 4 (3), e255. 10.1002/mco2.255 37090118 PMC10119582

[B29] Liu X. Zhang Z. Ruan J. Pan Y. Magupalli V. G. Wu H. (2016). Inflammasome-activated gasdermin D causes pyroptosis by forming membrane pores. Nature 535 (7610), 153–158. 10.1038/nature18629 27383986 PMC5539988

[B30] Liu Y. Y. Yu L. H. Zhang J. Xie D. J. Zhang X. X. Yu J. M. (2021c). Network pharmacology-based and molecular docking-based analysis of Suanzaoren decoction for the treatment of Parkinson's disease with sleep disorder. Biomed. Res. Int. 2021, 1752570. 10.1155/2021/1752570 34660782 PMC8519686

[B31] Liu Z. Roberts R. A. Lal-Nag M. Chen X. Huang R. Tong W. (2021b). AI-based language models powering drug discovery and development. Drug Discov. Today 26 (11), 2593–2607. 10.1016/j.drudis.2021.06.009 34216835 PMC8604259

[B32] Lu W. Q. Qiu Y. Li T. J. Tao X. Sun L. N. Chen W. S. (2009). Timosaponin B-II inhibits pro-inflammatory cytokine induction by lipopolysaccharide in BV2 cells. Arch. Pharm. Res. 32 (9), 1301–1308. 10.1007/s12272-009-1916-4 19784587

[B33] Luo W. Y. Gao L. Zhao D. D. Zhang L. Gao B. Lei G. (2023). Yunvjian improves glucose and insulin function in diabetic rats by regulating gastric emptying function. Evid. Based Complement. Altern. Med. 2023, 8551406. 10.1155/2023/8551406 PMC9867596 36691597

[B34] Lv J. Su M. Wang Y. Yang J. Liang Y. Chen L. (2024). Yunvjian decoction mitigates hyperglycemia in rats induced by a high-fat diet and streptozotocin via reducing oxidative stress in pancreatic beta cells. J. Ethnopharmacol. 327, 118045. 10.1016/j.jep.2024.118045 38479546

[B35] Minoru K. Miho F. Yoko S. Masayuki M. Mari I. (2023). KEGG for taxonomy-based analysis of pathways and genomes. Nucleic Acids Res. 51 (D1), D587–D592. 10.1093/nar/gkac963 36300620 PMC9825424

[B36] Mokrá D. (2020). Acute lung injury - from pathophysiology to treatment. Physiol. Res. 69 (Suppl. 3), S353–S366. 10.33549/physiolres.934602 33464919 PMC8603709

[B37] Nanchal R. S. Truwit J. D. (2018). Recent advances in understanding and treating acute respiratory distress syndrome. F1000Res 7. 10.12688/f1000research.15493.1 PMC6107983 30210781

[B38] Park B. K. So K. S. Ko H. J. Kim H. J. Kwon K. S. Kwon Y. S. (2018). Therapeutic potential of the rhizomes of anemarrhena asphodeloides and timosaponin A-III in an animal model of lipopolysaccharide-induced lung inflammation. Biomol. Ther. Seoul. 26 (6), 553–559. 10.4062/biomolther.2017.249 29925223 PMC6254648

[B39] Peng L. Wen L. Shi Q. F. Gao F. Huang B. Meng J. (2020). Scutellarin ameliorates pulmonary fibrosis through inhibiting NF-κB/NLRP3-mediated epithelial-mesenchymal transition and inflammation. Cell Death Dis. 11 (11), 978. 10.1038/s41419-020-03178-2 33188176 PMC7666141

[B40] Puttur F. Gregory L. G. Lloyd C. M. (2019). Airway macrophages as the guardians of tissue repair in the lung. Immunol. Cell Biol. 97 (3), 246–257. 10.1111/imcb.12235 30768869

[B41] Quang D. N. Harinantenaina L. Nishizawa T. Hashimoto T. Kohchi C. Soma G. (2006). Inhibition of nitric oxide production in RAW 264.7 cells by azaphilones from xylariaceous fungi. Biol. Pharm. Bull. 29 (1), 34–37. 10.1248/bpb.29.34 16394505

[B42] Rao Z. Zhu Y. Yang P. Chen Z. Xia Y. Qiao C. (2022). Pyroptosis in inflammatory diseases and cancer. Theranostics 12 (9), 4310–4329. 10.7150/thno.71086 35673561 PMC9169370

[B43] Ren M. S. Xie H. H. Ding Y. Li Z. H. Liu B. (2023). Er-xian decoction drug-containing serum promotes Mc3t3-e1 cell proliferation and osteogenic differentiation via regulating BK channel. J. Ethnopharmacol. 302 (PtA), 115887. 10.1016/j.jep.2022.115887 36328203

[B44] Ru J. Li P. Wang J. Zhou W. Li B. Huang C. (2014). TCMSP: a database of systems pharmacology for drug discovery from herbal medicines. J. Cheminform 6, 13. 10.1186/1758-2946-6-13 24735618 PMC4001360

[B45] Šerý O. Hlinecká L. Povová J. Bonczek O. Zeman T. Janout V. (2016). Arachidonate 5-lipoxygenase (ALOX5) gene polymorphism is associated with Alzheimer's disease and body mass index. J. Neurol. Sci. 362, 27–32. 10.1016/j.jns.2016.01.022 26944113

[B46] Shang L. Zhang M. Li J. Zhou F. Wang S. Chen L. (2024). Dachengqi decoction alleviates acute lung injury by suppressing HIF-1α-mediated glycolysis. J. Ethnopharmacol. 321, 117410. 10.1016/j.jep.2023.117410 37989425

[B47] Shi J. Zhao Y. Wang K. Shi X. Wang Y. Huang H. (2015). Cleavage of GSDMD by inflammatory caspases determines pyroptotic cell death. Nature 526, 660–665. 10.1038/nature15514 26375003

[B48] Soliman M. G. Mansour H. A. Hassan W. A. Shawky E. (2022). Protective effects of amoxicillin and probiotics on colon disorders in an experimental model of acute diverticulitis disease. Inflammopharmacology 30 (6), 2153–2165. 10.1007/s10787-022-01093-w 36318434 PMC9700596

[B49] Tang J. Xu L. Zeng Y. Gong F. (2021). Effect of gut microbiota on LPS-induced acute lung injury by regulating the TLR4/NF-kB signaling pathway. Int. Immunopharmacol. 91, 107272. 10.1016/j.intimp.2020.107272 33360370

[B50] Tang Y. S. Zhao Y. H. Zhong Y. Li X. Z. Pu J. X. Luo Y. C. (2019). Neferine inhibits LPS-ATP-induced endothelial cell pyroptosis via regulation of ROS/NLRP3/Caspase-1 signaling pathway. Inflamm. Res. 68, 727–738. 10.1007/s00011-019-01256-6 31172209

[B51] Thompson B. T. Chambers R. C. Liu K. D. (2017). Acute respiratory distress syndrome. N. Engl. J. Med. 377, 562–572. 10.1056/NEJMra1608077 28792873

[B52] Wang L. Hauenstein A. V. (2020). The NLRP3 inflammasome: mechanism of action, role in disease and therapies. Mol. Asp. Med. 76, 100889. 10.1016/j.mam.2020.100889 32859386

[B53] Wang L. Yang H. Qiao L. Liu J. Liao X. Huang H. (2022b). Ophiopogonin D inhibiting epithelial NF-κB signaling pathway protects against experimental colitis in mice. Inflammation 45 (4), 1720–1731. 10.1007/s10753-022-01655-8 35460395

[B54] Wang Y. Wang Y. Ma J. Li Y. Cao L. Zhu T. (2022a). YuPingFengSan ameliorates LPS-induced acute lung injury and gut barrier dysfunction in mice. J. Ethnopharmacol. 312, 116452. 10.1016/j.jep.2023.116452 37019161

[B55] Yao Q. W. Wang X. Y. Li J. C. Zhang J. (2019). Ophiopogon japonicus inhibits radiation-induced pulmonary inflammation in mice. Ann. Transl. Med. 7 (22), 622. 10.21037/atm.2019.11.01 31930023 PMC6944568

[B56] Ye Q. Lin B. Xu P. Zhang F. Wang N. Shou D. (2024). Yunvjian decoction attenuates lipopolysaccharide-induced periodontitis by suppressing NFκB/NLRP3/IL-1β pathway. J. Ethnopharmacol. 319 (Pt2), 117279. 10.1016/j.jep.2023.117279 37802377

[B57] Zhang Y. Li X. Grailer J. J. Wang N. Wang M. Yao J. (2016). Melatonin alleviates acute lung injury through inhibiting the NLRP3 inflammasome. J. Pineal Res. 60 (4), 405–414. 10.1111/jpi.12322 26888116

