# Supplementary material for: Yunvjian decoction attenuates lipopolysaccharide-induced acute lung injury by inhibiting NF-κB/NLRP3 pathway and pyroptosis
Source: Front Pharmacol. 2025 Jan 24;16:1430536. doi: 10.3389/fphar.2025.1430536 (PMC11802820; doi:10.3389/fphar.2025.1430536)
Supplement: Supplementary file 2 [file DataSheet7.docx]

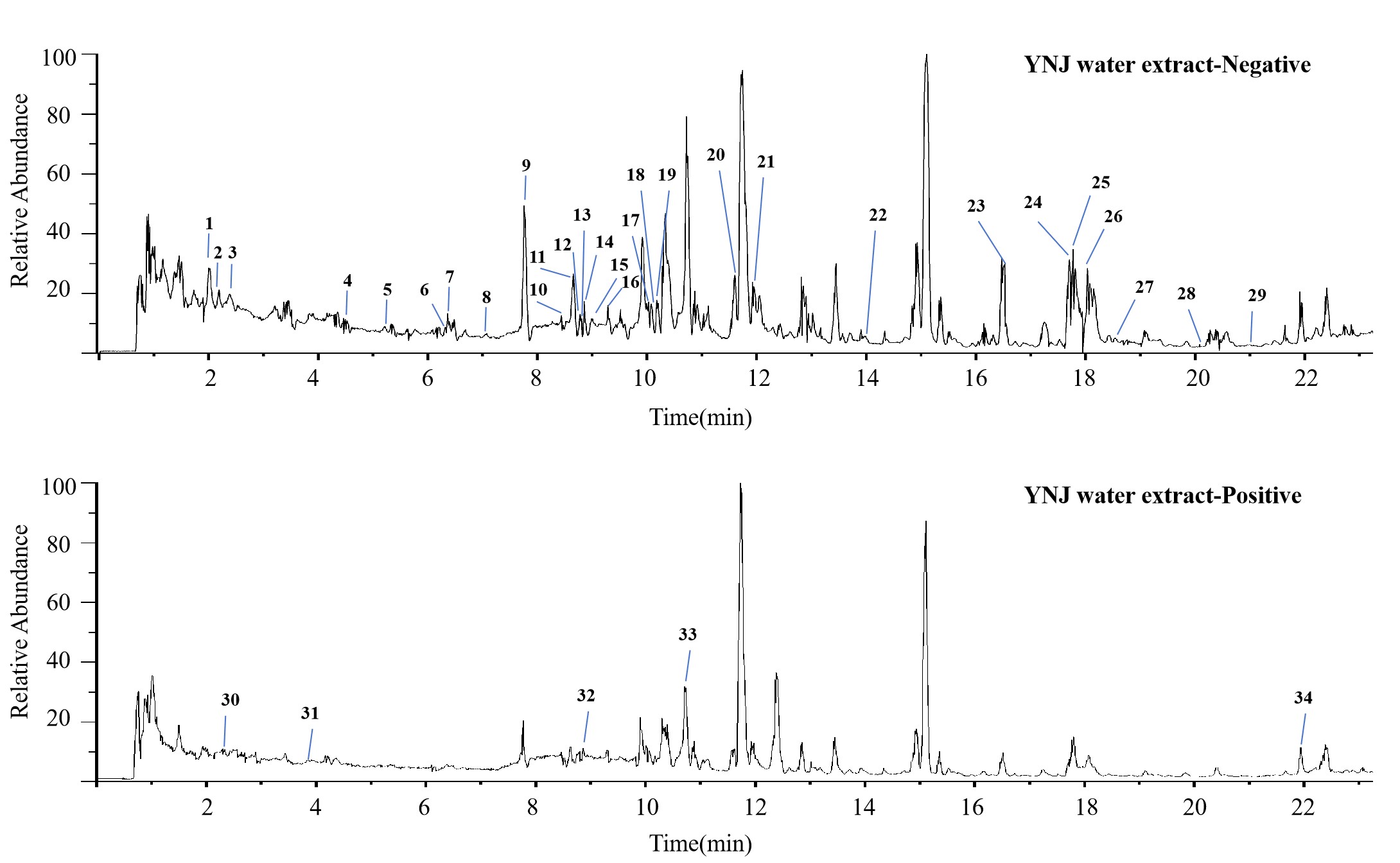


**Figure S7**

Total ion chromatograms of YNJ water extract in the positive-ion and negative-ion modes.
