# Supplementary material for: Yunvjian decoction attenuates lipopolysaccharide-induced acute lung injury by inhibiting NF-κB/NLRP3 pathway and pyroptosis
Source: Front Pharmacol. 2025 Jan 24;16:1430536. doi: 10.3389/fphar.2025.1430536 (PMC11802820; doi:10.3389/fphar.2025.1430536)
Supplement: Supplementary file 4 [file Table1.docx]

**Table S1** Primer sequences

| Genes | Forward (5'-3') | Reverse (5'-3') |
| --- | --- | --- |
| NLRP3 | TCTGTTCATTGGCTGCGGATGG | TGGTCCCTTCCTCACGGTCAC |
| ASC | TGGACAAGGCACGGGACCTATG | AGGGCAAGACGTGTACGAGTGG |
| Caspase-1 | TGGACAAGGCACGGGACCTATG | AGGGCAAGACGTGTACGAGTGG |
| IL-1β | TTCAGGCAGGCAGTATCACTCATTG | TGTCGTTGCTTGGTTCTCCTTGTAC |
| GAPDH | AATGGTGAAGGTCGGTGTGAACG | TCGCTCCTGGAAGATGGTGATGG |
