# Supplementary figures and images for: Yunvjian decoction attenuates lipopolysaccharide-induced acute lung injury by inhibiting NF-κB/NLRP3 pathway and pyroptosis
Source: Front Pharmacol. 2025 Jan 24;16:1430536. doi: 10.3389/fphar.2025.1430536 (PMC11802820; doi:10.3389/fphar.2025.1430536)

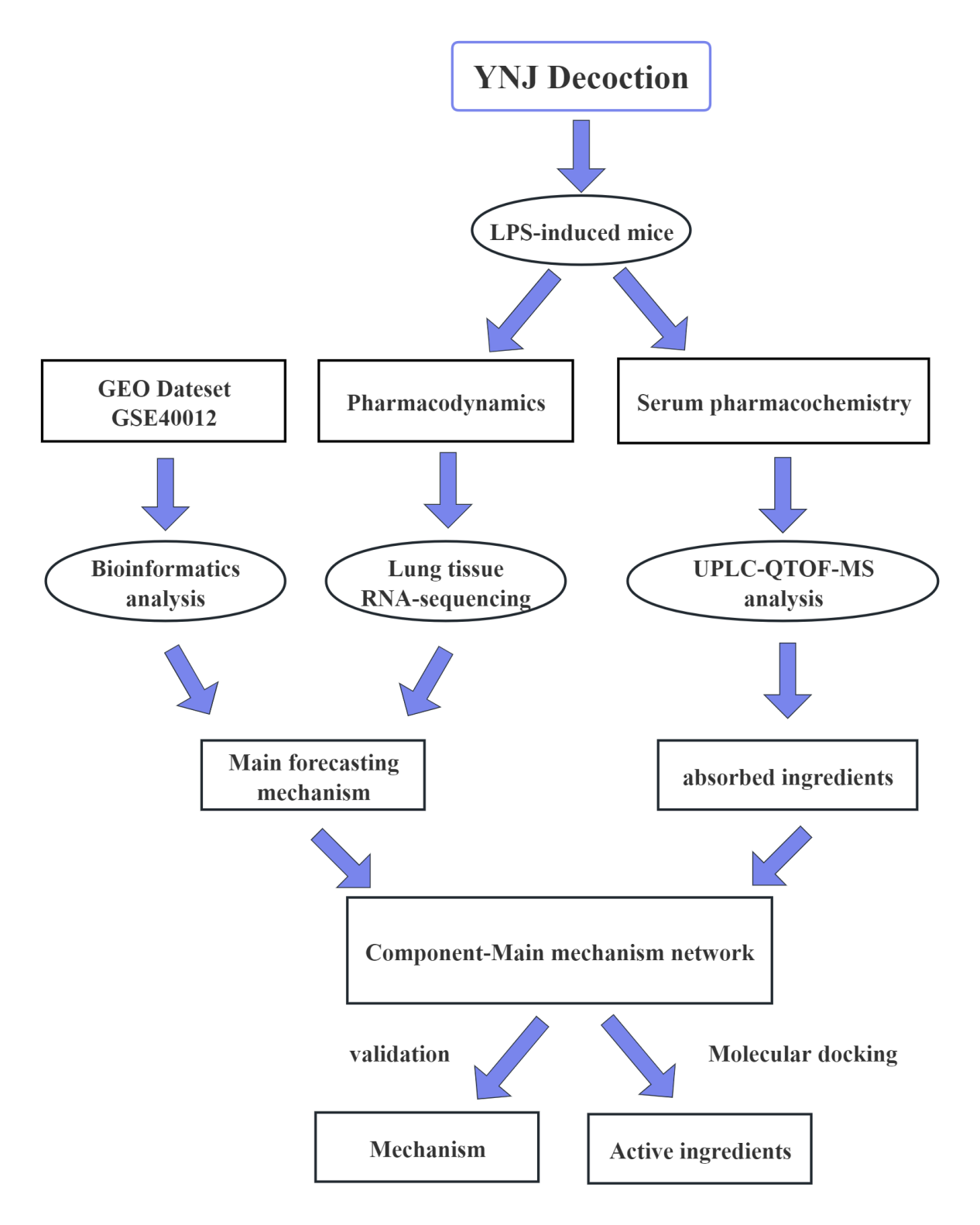


**Figure S9**

Schematic diagram of the pharmacological effects and mechanisms of YNJ against ALI

Supplement: Supplementary file 6 [file DataSheet9.docx]

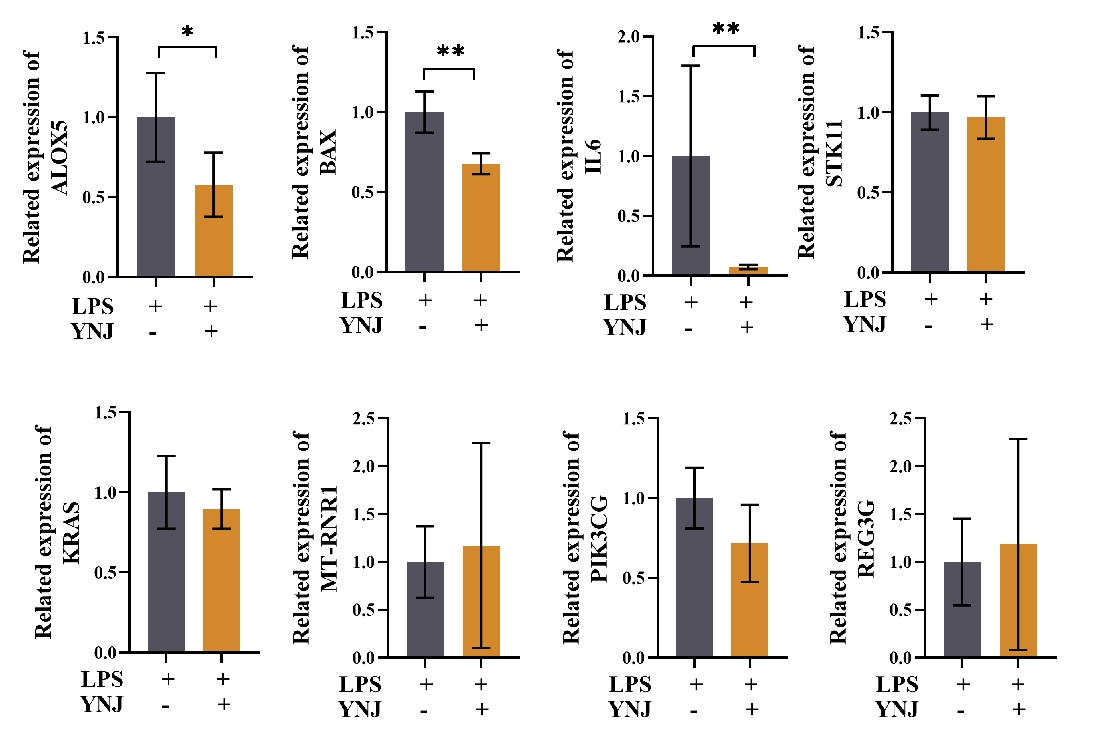


**Figure S1**

RNA sequencing revealed the effect of YNJ on ALI-related genes.

Supplement: Supplementary file 14 [file DataSheet1.docx]
