# Supplementary material for: Yunvjian decoction attenuates lipopolysaccharide-induced acute lung injury by inhibiting NF-κB/NLRP3 pathway and pyroptosis
Source: Front Pharmacol. 2025 Jan 24;16:1430536. doi: 10.3389/fphar.2025.1430536 (PMC11802820; doi:10.3389/fphar.2025.1430536)
Supplement: Supplementary file 8 [file DataSheet10.docx]

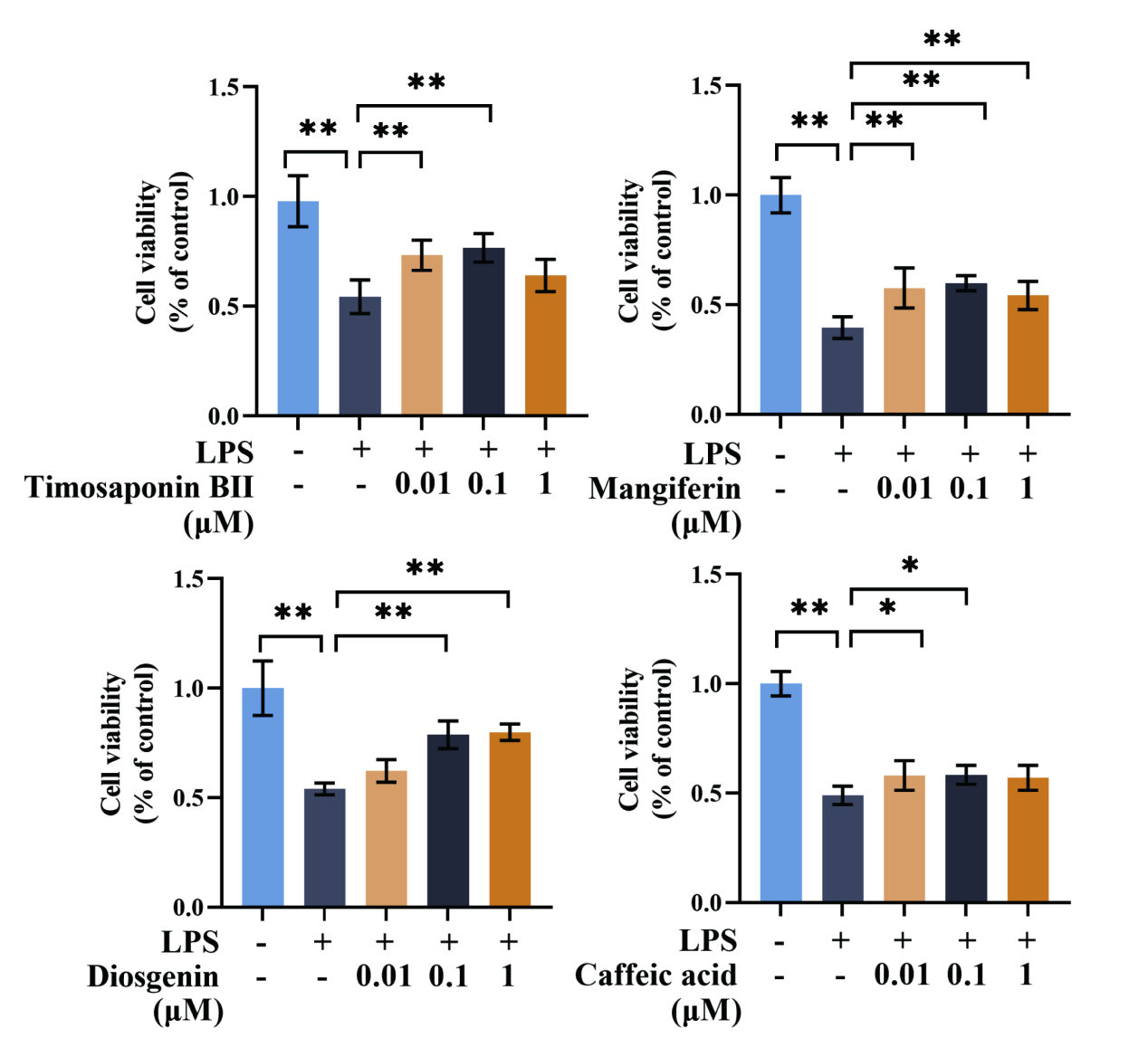


**Figure S10**

Effects of the predicted compounds on the cell viability of the LPS-induced MLE-12 cells. The data are presented as the mean ± SD. **P* < 0.05, ***P* < 0.01.
