# Supplementary material for: Yunvjian decoction attenuates lipopolysaccharide-induced acute lung injury by inhibiting NF-κB/NLRP3 pathway and pyroptosis
Source: Front Pharmacol. 2025 Jan 24;16:1430536. doi: 10.3389/fphar.2025.1430536 (PMC11802820; doi:10.3389/fphar.2025.1430536)
Supplement: Supplementary file 9 [file Table2.docx]

| **Table S2** Analysis and identification of YNJ water extract and mouse drug-containing serum prototype components | | | | | | | | | |
| --- | --- | --- | --- | --- | --- | --- | --- | --- | --- |
| No. | Time/min | Compound | Formula | Measured Mass (m/z) | Ion Addition | Mass  Error/ppm | MS/MS | *Origin | Detected in serum |
| 1 | 1.93 | Citric acid | C_6_H_8_O_7_ | 191.0200 | [M-H]- | 1.3 | 87.0089 | Rg | √ |
| 2 | 2.19 | Verbascose | C_30_H_52_O_26_ | 827.2679 | [M-H]- | 0.6 | 179.0562 | Rg | √ |
| 3 | 2.4 | Uridine | C_9_H_12_N_2_O_6_ | 243.0625 | [M-H]- | 1.0 | 111.0189 | Rg | √ |
| 4 | 4.51 | Melittoside | C_21_H_32_O_15_ | 523.1680 | [M-H]- | 2.2 | 89.0252 | Rg | √ |
| 5 | 5.36 | Gardoside | C_16_H_22_O_10_ | 373.1147 | [M-H]- | 1.7 | 149.0612;89.0251 | Rg | √ |
| 6 | 6.37 | Verbasoside | C_20_H_30_O_12_ | 461.1660 | [M-H]- | 1.0 | 135.0454; 161.0457; 315.1075 | Rg | × |
| 7 | 6.48 | loganic acid | C_16_H_24_O_10_ | 375.1306 | [M-H]- | 2.6 | 151.0765; 89.0240 | Rg | √ |
| 8 | 7.06 | Cistanoside F | C_21_H_28_O_13_ | 487.146 | [M-H]- | 1.6 | 179.0356 | Rg | × |
| 9 | 7.77 | Neomangiferin | C_25_H_28_O_16_ | 583.1318 | [M-H]- | 2.3 | 259.0244 | Aa | √ |
| 10 | 8.41 | Caffeic acid | C_9_H_8_O_4_ | 179.0362 | [M-H]- | 6.8 | 135.0452;133.0297 | Rg | √ |
| 11 | 8.62 | sec-Hydroxyaeginetic acid | C_15_H_24_O_5_ | 283.1554 | [M-H]- | 1.2 | 139.1129 | Rg | √ |
| 12 | 8.66 | Mangiferin | C_19_H_18_O_11_ | 421.0786 | [M-H]- | 2.3 | 259.0490; 301.0371; 343.0432 | Aa | √ |
| 13 | 8.86 | Purpureaside C | C_35_H_46_O_20_ | 785.251 | [M-H]- | 1.1 | 161.0244; 623.2205 | Rg | × |
| 14 | 8.86 | 4-O-Coumaroylquinic acid | C16H18O8 | 337.093 | [M-H]- | 1.1 | 119.0515; 173.0463 | Jk | × |
| 15 | 8.97 | Rehmapicroside | C_16_H_26_O_8_ | 345.1574 | [M-H]- | 5.4 | 183.1027 | Rg | √ |
| 16 | 9.31 | Cistanoside A | **C_36_H_48_O_20_** | 7990267 | [M-H]- | 2.0 | 605.2099 | Rg | × |
| 17 | 9.94 | Jionoside B1 | C_37_H_50_O_20_ | 813.2859 | [M-H]- | 4.5 | 175.0401 | Rg | √ |
| 18 | 10.20 | acteoside | C_29_H_36_O_15_ | 623.1980 | [M-H]- | 2.1 | 179.0358 | Rg | × |
| 19 | 10.33 | Timosaponin E1 | C_45_H_76_O_20_ | 935.4887 | [M-H]- | 3.2 | 161.0454 | Aa | √ |
| 20 | 11.72 | Timosaponin B-II | C_45_H_76_O_19_ | 919.4947 | [M-H]- | 4.2 | 757.4465 | Aa | √ |
| 21 | 11.95 | N-p-trans-Coumaroyltyramine | C_17_H_17_NO_3_ | 282.1140 | [M-H]- | 1.0 | 119.0640; 162.0573 | Aa | × |
| 22 | 14.00 | Anemarrhenasaponin F | C_39_H_64_O_15_ | 771.4170 | [M-H]- | 0.3 | 609.3821 | Aa | × |
| 23 | 16.52 | GinsenosideRo | C_48_H_76_O_19_ | 955.4934 | [M-H]- | 2.7 | 793.4366; 613.3746 | Ab | √ |
| 24 | 17.74 | Bidentatoside I | C_47_H_70_O_20_ | 953.4446 | [M-H]- | 6.1 | 909.4459 | Ab | √ |
| 25 | 17.80 | Anemarrhenasaponin I | C_39_H_66_O_14_ | 757.4374 | [M-H]- | 0.8 | 161.0472; 595.3873 | Aa | × |
| 26 | 18.07 | Zingibroside R1 | C_42_H_66_O_14_ | 793.4387 | [M-H]- | 0.9 | 631.3837 | Ab | √ |
| 27 | 18.55 | Achyranthoside C | C_46_H_70_O_19_ | 925.4440 | [M-H]- | -0.3 | 631.3878 | Ab | × |
| 28 | 20.27 | Hinokiresinol | C_17_H_16_O_2_ | 251.1097 | [M-H]- | 7.7 | 157.0659; 117.0342 | Aa | √ |
| 29 | 21.1 | OphiopogonanoneE | C_19_H_20_O_7_ | 359.114 | [M-H]- | 0.2 | 207.0256 | Jk | × |
| 30 | 2.31 | l-isoleucine | C_6_H_13_NO_2_ | 132.1016 | [M+H]+ | -2.0 | 86.0961 | Ab | √ |
| 31 | 3.92 | Phenylalanine | C_9_H_11_NO_2_ | 166.0858 | [M+H]+ | -2.5 | 103.0537; 91.0542 | Ab | √ |
| 32 | 9.88 | Vitexin | C_21_H_20_O_10_ | 433.1129 | [M+H]+ | -0.1 | 313.0707 | Aa | √ |
| 33 | 10.73 | Diosgenin | C_27_H_42_O_3_ | 415.3185 | [M+H]+ | -5.3 | 273.2221 | Ab | √ |
| 34 | 21.94 | Sarsasapogenin | C_27_H_44_O_3_ | 417.3324 | [M+H]+ | -9.5 | 399.3249 | Ab | √ |

*: Aa: *Anemarrhena asphodeloides* Bunge.; Ab: *A*. *Achyranthes bidentata Blume*; Rg:*Rehmannia glutinosa* (Gaertn.) DC.; Jk:*O*. *japonicus* (Thunb.) Ker Gawl.
