# Supplementary material for: Yunvjian decoction attenuates lipopolysaccharide-induced acute lung injury by inhibiting NF-κB/NLRP3 pathway and pyroptosis
Source: Front Pharmacol. 2025 Jan 24;16:1430536. doi: 10.3389/fphar.2025.1430536 (PMC11802820; doi:10.3389/fphar.2025.1430536)
Supplement: Supplementary file 10 [file DataSheet2.docx]

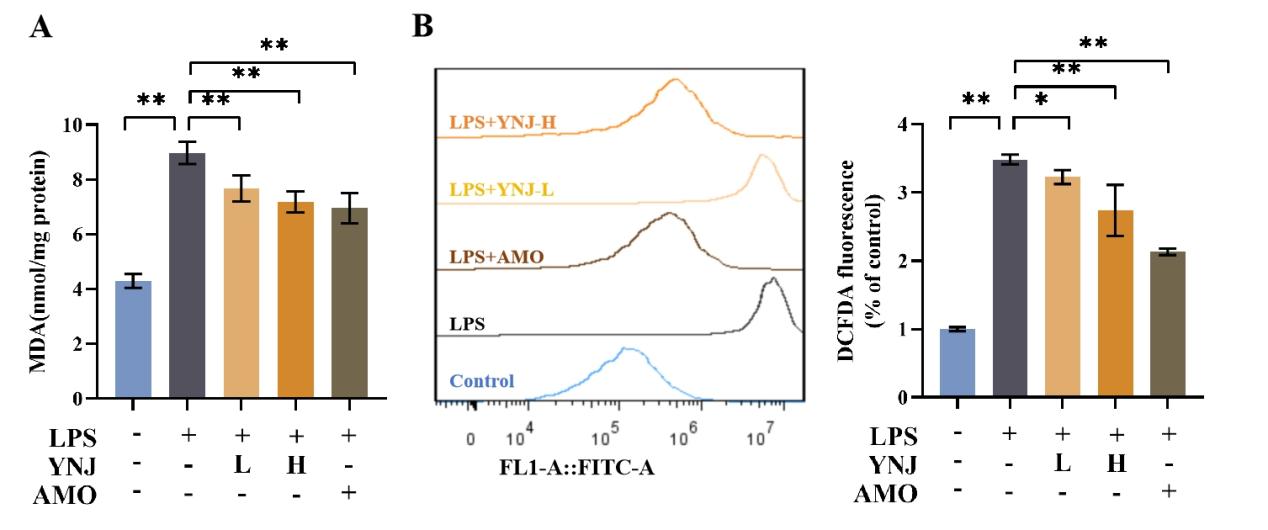


**Figure S2**

YNJ reduced oxidative stress in LPS-induced MLE-12 cells. (A) MDA contents. (B) ROS levels. Data are presented as mean ± SD. **P* < 0.05, ***P* < 0.01
