# Supplementary material for: Yunvjian decoction attenuates lipopolysaccharide-induced acute lung injury by inhibiting NF-κB/NLRP3 pathway and pyroptosis
Source: Front Pharmacol. 2025 Jan 24;16:1430536. doi: 10.3389/fphar.2025.1430536 (PMC11802820; doi:10.3389/fphar.2025.1430536)
Supplement: Supplementary file 11 [file DataSheet8.docx]

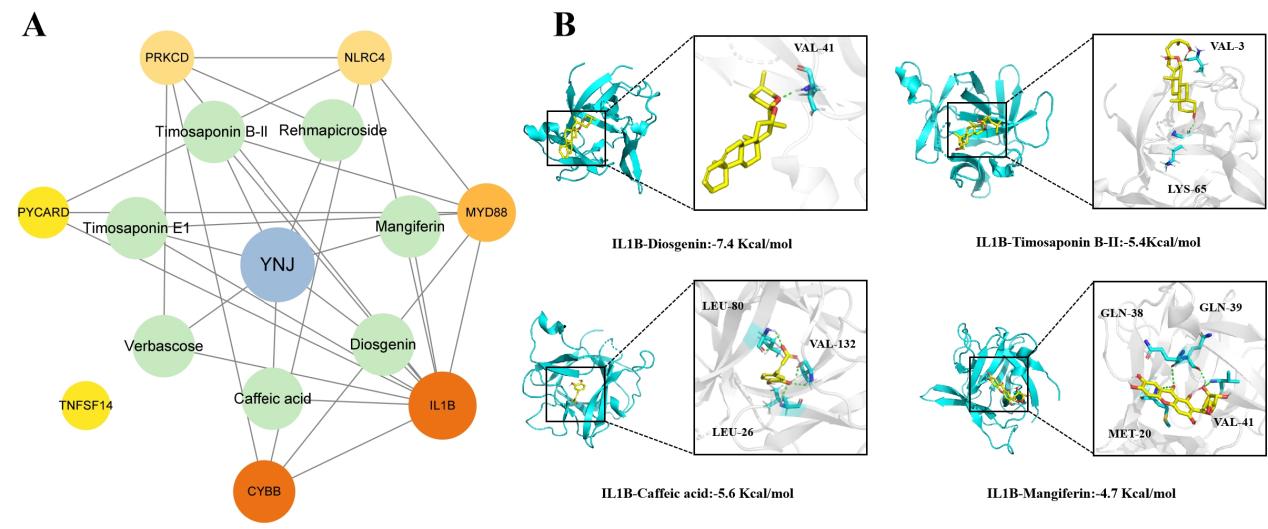


**Figure S8**

Prediction of the ALI targets of YNJ ingredients. (A) Compound-NF-κB/NLRP3-related target network of YNJ. (B) Molecular docking of IL1B with its related compounds.
