# Supplementary material for: Yunvjian decoction attenuates lipopolysaccharide-induced acute lung injury by inhibiting NF-κB/NLRP3 pathway and pyroptosis
Source: Front Pharmacol. 2025 Jan 24;16:1430536. doi: 10.3389/fphar.2025.1430536 (PMC11802820; doi:10.3389/fphar.2025.1430536)
Supplement: Supplementary file 12 [file DataSheet12.docx]

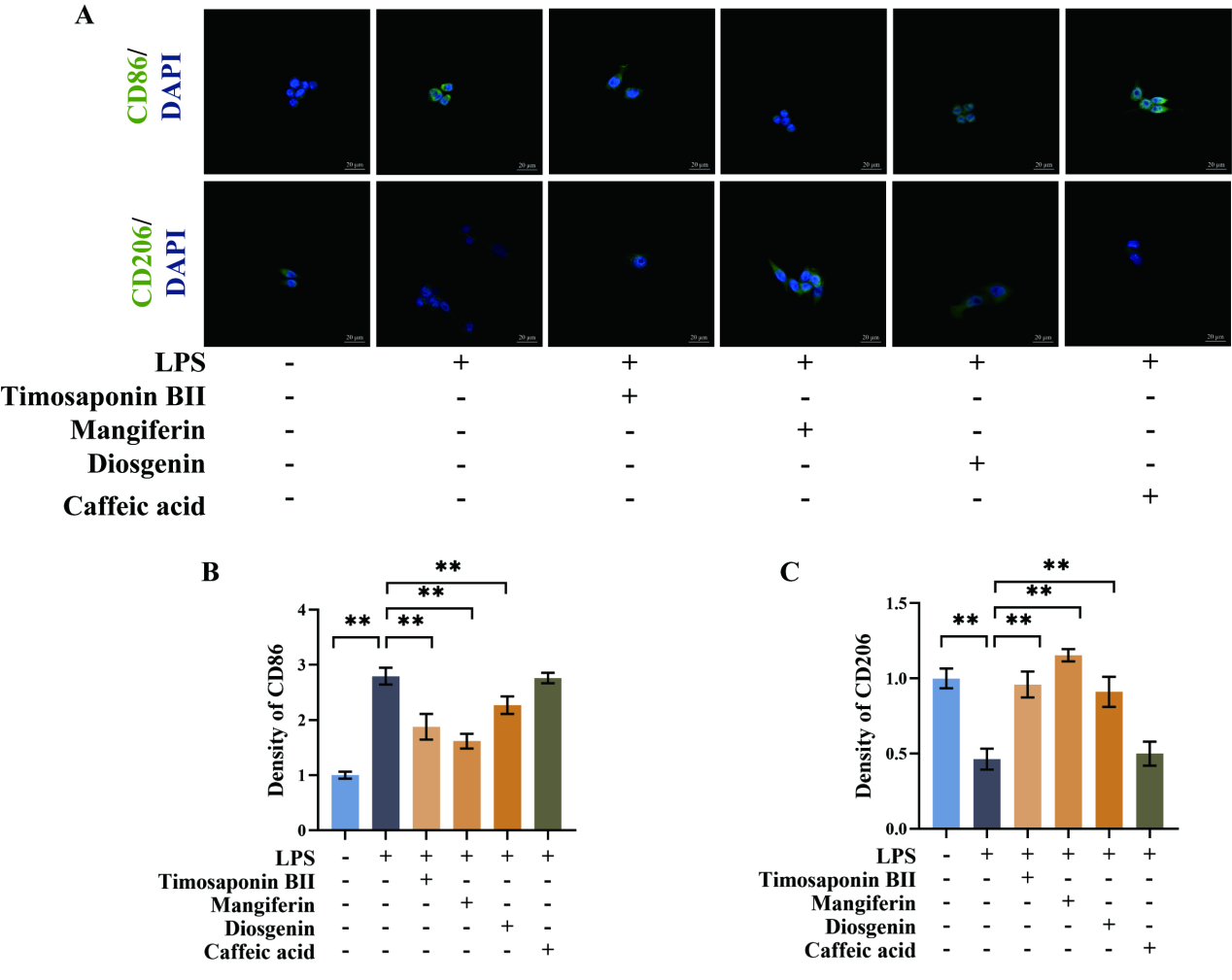


**Figure S12**

Effects of the predicted components on the M2-type polarization of macrophages. (A) Immunofluorescence images of CD86 and CD206. (B-C) Statistical analysis of CD86 and CD206. The data are presented as the mean ± SD (n=6). ***P* < 0.01.
